# Magnetic Resonance Imaging–Guided vs Computed Tomography–Guided Stereotactic Body Radiotherapy for Prostate Cancer: The MIRAGE Randomized Clinical Trial

**DOI:** 10.1001/jamaoncol.2022.6558

**Published:** 2023-01-12

**Authors:** Amar U. Kishan, Ting Martin Ma, James M. Lamb, Maria Casado, Holly Wilhalme, Daniel A. Low, Ke Sheng, Sahil Sharma, Nicholas G. Nickols, Jonathan Pham, Yingli Yang, Yu Gao, John Neylon, Vincent Basehart, Minsong Cao, Michael L. Steinberg

**Affiliations:** 1 Department of Radiation Oncology, University of California, Los Angeles; 2 Department of Urology, University of California, Los Angeles; 3 Statistics Core, Department of Medicine, University of California, Los Angeles

## Abstract

**Importance:**

Magnetic resonance imaging (MRI) guidance offers multiple theoretical advantages in the context of stereotactic body radiotherapy (SBRT) for prostate cancer. However, to our knowledge, these advantages have yet to be demonstrated in a randomized clinical trial.

**Objective:**

To determine whether aggressive margin reduction with MRI guidance significantly reduces acute grade 2 or greater genitourinary (GU) toxic effects after prostate SBRT compared with computed tomography (CT) guidance.

**Design, Setting, and Participants:**

This phase 3 randomized clinical trial (MRI-Guided Stereotactic Body Radiotherapy for Prostate Cancer [MIRAGE]) enrolled men aged 18 years or older who were receiving SBRT for clinically localized prostate adenocarcinoma at a single center between May 5, 2020, and October 1, 2021. Data were analyzed from January 15, 2021, through May 15, 2022. All patients had 3 months or more of follow-up.

**Interventions:**

Patients were randomized 1:1 to SBRT with CT guidance (control arm) or MRI guidance. Planning margins of 4 mm (CT arm) and 2 mm (MRI arm) were used to deliver 40 Gy in 5 fractions.

**Main Outcomes and Measures:**

The primary end point was the incidence of acute (≤90 days after SBRT) grade 2 or greater GU toxic effects (using Common Terminology Criteria for Adverse Events, version 4.03 [CTCAE v4.03]). Secondary outcomes included CTCAE v4.03–based gastrointestinal toxic effects and International Prostate Symptom Score (IPSS)–based and Expanded Prostate Cancer Index Composite-26 (EPIC-26)–based outcomes.

**Results:**

Between May 2020 and October 2021, 156 patients were randomized: 77 to CT (median age, 71 years [IQR, 67-77 years]) and 79 to MRI (median age, 71 years [IQR, 68-75 years]). A prespecified interim futility analysis conducted after 100 patients reached 90 or more days after SBRT was performed October 1, 2021, with the sample size reestimated to 154 patients. Thus, the trial was closed to accrual early. The incidence of acute grade 2 or greater GU toxic effects was significantly lower with MRI vs CT guidance (24.4% [95% CI, 15.4%-35.4%] vs 43.4% [95% CI, 32.1%-55.3%]; *P* = .01), as was the incidence of acute grade 2 or greater gastrointestinal toxic effects (0.0% [95% CI, 0.0%-4.6%] vs 10.5% [95% CI, 4.7%-19.7%]; *P* = .003). Magnetic resonance imaging guidance was associated with a significantly smaller percentage of patients with a 15-point or greater increase in IPSS at 1 month (6.8% [5 of 72] vs 19.4% [14 of 74]; *P* = .01) and a significantly reduced percentage of patients with a clinically significant (≥12-point) decrease in EPIC-26 bowel scores (25.0% [17 of 68] vs 50.0% [34 of 68]; *P* = .001) at 1 month.

**Conclusions and Relevance:**

In this randomized clinical trial, compared with CT-guidance, MRI-guided SBRT significantly reduced both moderate acute physician-scored toxic effects and decrements in patient-reported quality of life. Longer-term follow-up will confirm whether these notable benefits persist.

**Trial Registration:**

ClinicalTrials.gov Identifier: NCT04384770

## Key Points

**Question:**

Does the aggressive margin reduction allowed by magnetic resonance imaging (MRI) guidance vs computed tomography guidance allow a significant reduction in toxic effects following stereotactic body radiotherapy for prostate cancer?

**Findings:**

In this phase 3 randomized clinical trial of 156 patients with prostate cancer, MRI guidance significantly reduced acute moderate physician-scored genitourinary and gastrointestinal toxic effects and led to smaller decrements in patient-reported urinary and bowel function.

**Meaning:**

The results of this study suggest a significant advantage to MRI-guided radiotherapy, which allows aggressive margin reduction, for patients with prostate cancer.

## Teaser

This randomized clinical trial assesses genitourinary toxic effects after magnetic resonance imaging (MRI)–guided vs computed tomography (CT)–guided stereotactic body radiotherapy for prostate cancer.

## Introduction

Emerging clinical trial data have demonstrated stereotactic body radiotherapy (SBRT), a form of radiation in which large daily doses are delivered with high precision in generally 5 or fewer treatments, to be a curative option for very low-risk through very high-risk prostate cancer.^1,2,3,4,5^ Traditionally, SBRT has been delivered using linear accelerators (LINACs) relying on computed tomography (CT)–based planning techniques and the use of either planar x-ray or cone-beam CT images to guide radiation delivery. While generally well tolerated, both acute and late toxic effects can manifest as urinary, bowel, and sexual dysfunction and contribute to treatment-related burden.^6,7,8,9^

Magnetic resonance imaging (MRI)–guided LINACs have recently become commercially available and offer several theoretical advantages.^10,11^ First, MRI-guided LINACs can monitor prostate motion directly rather than relying on fiducial markers that are proxies for prostatic motion and require an invasive procedure to place. The improved soft-tissue contrast from an on-board MRI also improves the accuracy of alignment prior to radiation.^12^ Furthermore, residual error from the fusion of diagnostic MRI images to planning CT scans for contour delineation can be minimized with the direct use of MRI images for contouring; even if a diagnostic MRI were to be fused, this would be an MRI-MRI fusion with minimal uncertainty.^11,13^ In concert, these advantages of MRI guidance should allow reductions in the planning target volume (PTV) margins, which are typically used to ensure adequate target dosing.^14^ The high-dose regions of the PTV often overlap portions of the bladder, rectum, and other nearby structures and contribute to toxicity.^15,16,17,18^

To our knowledge, the theoretical advantages of MRI guidance have not been rigorously studied in a randomized clinical trial. Given the increased resources needed for MRI-guided radiotherapy,^10,19,20^ it is imperative to establish that it offers a tangible benefit for patients. We therefore conducted the CT-Guided Body Radiation Therapy and MRI-Guided Stereotactic Body Radiation Therapy for Prostate Cancer (MIRAGE) phase 3 randomized clinical trial (NCT04384770), in which we aimed to demonstrate that aggressive PTV margin reduction with MRI-guided radiotherapy reduces acute toxic effects following SBRT for localized prostate cancer.

## Methods

### Study Design and Participants

In this nonblinded, single-center, phase 3 randomized clinical trial, men aged 18 years or older with histologically confirmed, clinically localized prostate adenocarcinoma (ie, cN0M0 on conventional imaging) were enrolled between May 5, 2020, and October 1, 2021. Key eligibility criteria included a standard-of-care workup.^5^ Exclusion criteria included presence of non-MRI–compatible electronic devices or foreign objects and severe claustrophobia. The study was approved by the institutional review board of the University of California, Los Angeles, and written informed consent was obtained from all patients. The trial protocol is provided in Supplement 1.^21^ This study followed the Consolidated Standards of Reporting Trials (CONSORT) reporting guideline.

### Randomization

We randomly assigned (1:1) eligible patients to receive either CT-guided SBRT or MRI-guided SBRT using a computer-generated randomization list with permuted blocks of 6 (with the block size known only to the statistician [H.W.] until completion of the study). Randomization was stratified by the baseline International Prostate Symptom Score (IPSS) (≤15 or >15)^22^ and prostate gland volume (≤50 cc or >50 cc) as determined by a diagnostic MRI. Neither the patient nor the treating physician was blinded to the allocation.

### Radiotherapy

Magnetic resonance imaging–guided SBRT was delivered using the MRIdian LINAC (ViewRay, Inc), whereas CT-guided SBRT was delivered using TrueBeam (Varian Medical Systems, Inc) or Novalis Tx (Brainlab AG and Varian Medical Systems, Inc). All patients underwent CT simulation. For patients treated with CT guidance, fiducial markers were placed transperineally on the day of the scan; for patients treated with MRI guidance, fiducials were not placed, but a free-breathing 0.35T MRI simulation scan using a true fast imaging with steady state precession sequence was obtained.^23^ For patients in either arm, a full bladder and empty rectum were required for the simulation sessions and prior to each treatment. For all patients, fused 1.5T to 3T diagnostic MRIs were used to assist in contouring. For patients in the MRI arm, additional sequences were also obtained during the 0.35T MRI simulation scan for urethral delineation, as described previously.^24^

The clinical target volume was defined as the prostate and the proximal 1 cm of the seminal vesicle for all patients. This was expanded by 4 mm isotropically (CT guidance) or 2 mm isotropically (MRI guidance) to form the PTV. The 4-mm margin in the CT arm was based on a prior analysis of the margin necessary to account for motion as well as setup uncertainty in the context of CT-guided SBRT.^25^ The PTV was prescribed a dose of 40 Gy in 5 fractions, such that 95% of the PTV received this dose while abiding by organ-at-risk constraints (trial protocol in Supplement 1). Elective nodal radiotherapy (25 Gy in 5 fractions), a simultaneous integrated boost to the dominant intraprostatic lesion (42 Gy in 5 fractions), and a simultaneous integrated boost to a pelvic node deemed to be involved with metastatic disease on advanced functional imaging (35 Gy in 5 fractions) were allowed per investigator discretion. Androgen-deprivation therapy with or without a nonsteroidal antiandrogen or a second-generation hormonal therapy agent was allowed per investigator discretion.

Computed tomography–guided SBRT was delivered with volumetric-modulated arc therapy, whereas MRI-guided SBRT was delivered with step-and-shoot, intensity-modulated radiotherapy. Fractions were delivered every other day. For CT-guided SBRT, a cone-beam CT was obtained prior to each fraction to assess anatomic features and followed by planar imaging to align to fiducial markers; SBRT then commenced without further intrafraction monitoring given a total delivery time of less than 4 minutes. For MRI-guided SBRT, the prostate was aligned prior to each fraction with a true fast imaging with steady state precession MRI. During SBRT, cine MRI was obtained 4 times per second in the sagittal plane.^26^ If greater than 10% of the prostate volume moved outside a 3-mm gating boundary placed around the prostate, an automatic beam-hold would be initiated.

### Outcomes

The primary outcome was the incidence of acute genitourinary (GU) grade 2 or greater toxic effects scored on the Common Terminology Criteria for Adverse Events, version 4.03 (CTCAE v4.03)^27^ scale. This was evaluated from the start of SBRT to 90 days or fewer after SBRT. Secondary outcomes included acute CTCAE v4.03 gastrointestinal (GI) grade 2 or greater toxic effects and changes in IPSS^22^ and Expanded Prostate Cancer Index Composite-26 (EPIC-26).^28^ Clinically relevant decrement in EPIC-26 domains was defined as greater than 18, 14, 12, and 24 points for urinary incontinence, urinary irritative or obstructive, bowel, and sexual domains, respectively.^29^ A 15-point or greater increase in IPSS was considered clinically relevant. Patients were evaluated for toxic effects and completed IPSS and EPIC-26 questionnaires at baseline and at each scheduled follow-up, including at 1 month and 3 months for evaluation of the primary outcome.

### Statistical Analysis

The primary end point for this study was the incidence of acute grade 2 or greater GU toxic effects. The study was initially designed to detect a 14% reduction in acute toxic effects, from 29% to 15%, based on data from prior studies.^1,30^ An overall sample size of 300 patients (150 per arm) was estimated to provide 83.7% power to detect an absolute risk reduction of 14% with a 1-sided, 2-sample Z test at a *P* = .025 significance level. A prespecified interim analysis for futility was conducted once 100 patients reached 90 or more days after SBRT. Conditional power was assessed, and sample size was reestimated. The Pocock α-spending function was used to divide the α between the 2 analyses, providing α = .03 for the interim analysis and α = .019 for the final analysis. End points were summarized using frequencies and percentages; exact binomial proportion 95% CIs were also computed. To determine whether there was a difference in the proportion of grade 2 or greater GU toxic effects between CT and MRI guidance, the χ^2^ test was used; Fisher exact test was used to analyze grade 2 or greater GI toxic effects due to the low event rate. Subgroup analyses for IPSS (≤15 vs >15) and gland volume (≤50 cc vs >50 cc) were conducted for both end points in a similar manner, with interaction tests planned. Two-sided *P* values <.025 were considered statistically significant. As a post hoc exploratory analysis, logistic regression was used to develop multivariate prediction models for the primary end point. Candidate variables were selected using a combination of statistical significance in the univariate analyses as well as clinical expertise. Adjusted odds ratios and area under the receiver operating characteristic curve were calculated. The penalized regression method least absolute shrinkage and selection operator (LASSO) using cross-validation to select the tuning parameter was also used to further identify the subset of variables that were predictive of the primary end point. Two-sided *P* values <.05 were considered statistically significant. Differences in longitudinal changes (at 1 month and 3 months) for patient-reported outcomes were compared using the Mann-Whitney test. Proportions of patients with clinically relevant decreases (or, in the case of IPSS, increases) in patient-reported outcome scores at 1 month and 3 months and for the worst of the 2 were compared using the χ^2^ test. The *P* value threshold for significance was set at 0.025 given the superiority design of the trial. The primary end point was analyzed using a complete case analysis framework. No imputation of missing data was performed. All statistical analyses were conducted using SAS, version 9.4 (SAS Institute Inc). Data were analyzed from January 15, 2021, through May 15, 2022.

## Results

### Baseline Patient and Treatment Characteristics

**Figure 1.**
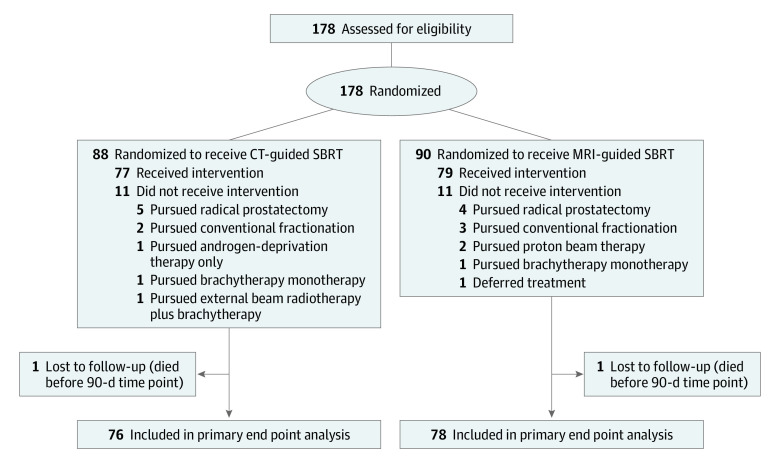
CONSORT Diagram CT indicates computed tomography; MRI, magnetic resonance imaging; SBRT, stereotactic body radiotherapy.

Between May 2020 and October 2021, 178 patients (88 in the CT arm and 90 in the MRI arm) were randomized. Eleven patients in each arm ultimately pursued a treatment other than SBRT, such that 156 patients received SBRT as randomized: 77 in the CT guided SBRT arm (median age, 71 years [IQR, 67-77 years]) and 79 in the MRI guided SBRT arm (median age, 71 years [IQR, 68-75 years]). A prespecified interim analysis was conducted on October 1, 2021, after 100 patients (51 patients in the CT guidance arm and 49 patients in the MRI guidance arm) were evaluable for the primary end point. The incidence of acute grade 2 or greater GU toxic effects was significantly reduced in men receiving MRI-guided SBRT compared with men receiving CT-guided SBRT (24 of 51 [47.1%] vs 11 of 49 [22.4%]; *P* = .01). Thus, a revised power calculation was performed, and it was concluded that a conditional power of 89% could be maintained with only 154 patients. By this time, 156 patients had already been treated, and the trial was closed for further accrual. The CONSORT diagram is shown in Figure 1; baseline patient and treatment characteristics are shown in Table 1. Overall, of 156 patients, 29 (19%) had favorable risk disease, 69 (44%) had placement of a rectal hydrogel spacer, 37 (24%) received nodal radiation, 41 (26%) received a simultaneous integrated boost to the dominant lesion, and 106 (68%) received hormonal therapy, with no significant differences between arms for any of these parameters. Treatment and dosimetric parameters are shown in eTables 1 and 2 in Supplement 2. Median postimaging delivery times were 1133 seconds (IQR, 1109-1289 seconds) with MRI guidance vs 232 seconds (IQR, 198-272 seconds) with CT guidance. There was no significant difference in clinical target volumes between the 2 arms.

**Table 1.** Baseline Characteristics

Characteristic	Patients (N = 156)^a^	
	CT arm (n = 77)	MRI arm (n = 79)
Age, median (IQR)	71 (67-77)	71 (68-75)
Risk group		
Imaging N0^b^		
Favorable intermediate	15 (19)	14 (18)
Unfavorable intermediate	25 (32)	40 (51)
High risk	21 (27)	15 (19)
Very high risk	9 (12)	5 (6)
Imaging N+^b^	7 (9)	5 (6)
Clinical T category 3 or 4^b^	1 (1)	4 (5)
Extracapsular extension on MRI	21 (27)	19 (25)
Seminal vesicle invasion on MRI	11 (14)	11 (14)
ADT use	57 (74)	49 (62)
Nodal radiation	19 (25)	18 (23)
GTV boost	22 (29)	19 (24)
Rectal spacer	32 (42)	37 (47)
Prior TURP or HOLEP	3 (4)	5 (6)
Prostate size, median (IQR), mL	41 (33-59)	39 (30-54)
IPSS, median (IQR)	6.0 (3.0-11.0)	7.0 (4.0-12.5)
Urinary medications at baseline	27 (35)	30 (38)
Baseline GI comorbidity	18 (23)	12 (15)
Hip replacement	3 (4)	6 (8)

Abbreviations: ADT, androgen-deprivation therapy; CT, computed tomography; GI, gastrointestinal; GTV, gross tumor volume; HOLEP, holmium laser enucleation of the prostate; IPSS, International Prostate Symptom Score; MRI, magnetic resonance imaging; TURP, transurethral vapor resection of the prostate.

^a^ Data are presented as number (percentage) of patients unless otherwise indicated.

^b^ According to American Joint Committee on Cancer 8th edition criteria.

### Physician-Scored Toxic Effects

**Figure 2.**
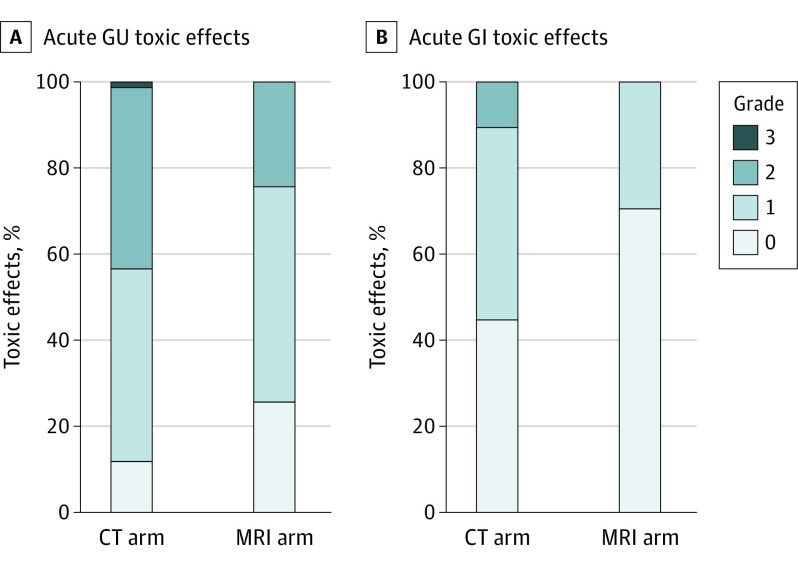
Rates of Acute Genitourinary (GU) and Gastrointestinal (GI) Toxic Effects All toxic effects were scored based on the Common Terminology Criteria for Adverse Events, version 4.03 scale. CT indicates computed tomography; MRI, magnetic resonance imaging.

Two patients (1 in the CT guidance arm and 1 in the MRI guidance arm) died due to COVID-19–related complications prior to the 90-day time window following SBRT, resulting in 154 analyzable patients (76 in the CT guidance arm and 78 in the MRI guidance arm) for a complete case analysis. Rates of toxic effects are shown in Table 2 and Figure 2. Rates of acute grade 2 or greater GU toxic effects were significantly lower with MRI vs CT guidance (19 of 78 [24.4%; 95% CI, 15.4%-35.4%] vs 33 of 76 [43.4%; 95% CI, 32.1%-55.3%]; *P* = .01). Sensitivity analyses were performed including the 2 additional patients who died prior to the 90-day end point, varying their toxic effects outcome; results were unchanged (eTable 3 in Supplement 2). On a post hoc exploratory multivariable analysis, including all the candidate variables of trial arm, age, IPSS, prostate volume, rectal spacer use, pelvic lymph node radiation, and use of a simultaneous integrated boost dose to gross disease, trial arm remained associated with a 60% reduction in odds of grade 2 or greater GU toxic effects (odds ratio, 0.4; 95% CI, 0.2-0.8; *P* = .02) (eTable 4 in Supplement 2). The penalized regression LASSO approach resulted in all variables remaining in the model except for prostate volume and rectal spacer use. Rates of acute grade 2 or greater GI toxic effects were also significantly lower with MRI guidance vs CT guidance (0 of 78 [0.0%; 95% CI, 0.0%-4.6%] vs 8 of 76 [10.5%; 95% CI, 4.7%-19.7%]; *P* = .003). As the absolute event rate for grade 2 or greater GI toxic effects was less than 10, no multivariable analysis was performed. Subgroup analyses for toxic effects within prespecified strata and univariate analyses are shown in eTables 5 and 6 in Supplement 2.

**Table 2.** Incidence of Acute Highest-Grade Treatment-Related Toxic Effects 3 Months or Less After SBRT^a^

Adverse event	Patients, No. (%) (N = 154)								*P* value^b^
	CT-guided SBRT (n = 76)				MRI-guided SBRT (n = 78)				
	Grade 1	Grade 2	Grade 3	Grade ≥2	Grade 1	Grade 2	Grade 3	Grade ≥2	
**Genitourinary**									
Any^c^	34 (44.7)	32 (42.1)	1 (1.3)	33 (43.4)	39 (50.0)	19 (24.4)	0	19 (24.4)	.006
Cystitis	2 (2.6)	2 (2.6)	0	2 (2.6)	0	0	0	0	.12
Hematuria	1 (1.3)	1 (1.3)	0	1 (1.3)	2 (2.6)	1 (1.3)	0	1 (1.3)	.50
Urinary frequency	32 (42.1)	24 (31.6)	0	24 (31.6)	28 (35.9)	12 (15.4)	0	12 (15.4)	.01
Urinary incontinence	9 (11.8)	3 (3.9)	0	3 (3.9)	4 (5.1)	2 (2.6)	0	2 (2.6)	.34
Urinary retention	10 (13.2)	20 (26.3)	1 (1.3)	21 (27.6)	7 (9.0)	9 (11.5)	0	9 (11.5)	.006
Urinary tract infection	0	0	0	0	0	0	0	0	.50
Urinary urgency	20 (26.3)	9 (11.8)	0	9 (11.8)	19 (24.4)	5 (6.4)	0	5 (6.4)	.14
Dysuria	9 (11.8)	5 (6.6)	0	5 (6.6)	1 (1.3)	5 (6.4)	0	5 (6.4)	.50
**Gastrointestinal**									
Any^c^	34 (44.7)	8 (10.5)	0	8 (10.5)	23 (29.5)	0	0	0	.001
Colitis	1 (1.3)	2 (2.6)	0	2 (2.6)	0	0	0	0	.12
Constipation	3 (3.9)	0	0	0	3 (3.8)	0	0	0	.50
Diarrhea	22 (28.9)	5 (6.6)	0	5 (6.4)	15 (19.2)	0	0	0	.01
Nausea	0	0	0	0	0	0	0	0	.50
Proctitis	15 (19.7)	5 (6.6)	0	5 (6.4)	9 (11.5)	0	0	0	.01
GI hemorrhage	4 (5.3)	3 (3.9)	0	3 (3.8)	1 (1.3)	0	0	0	.06
Rectal pain	2 (2.6)	2 (2.6)	0	2 (2.6)	1 (1.3)	0	0	0	.12
**Sexual**									
Any^c^	2 (2.6)	0	0	0	0	0	0	0	.50
Erectile dysfunction	2 (2.6)	0	0	0	0	0	0	0	.50

Abbreviations: CT, computed tomography; MRI, magnetic resonance imaging; SBRT, stereotactic body radiotherapy.

^a^ Toxic effects were graded according to Common Terminology Criteria for Adverse Events, version 4.03.

^b^ *P* values compare grade 2 or more toxic effects between the CT-guided and the MRI-guided SBRT arms, using χ^2^ or Fisher exact test when appropriate. One-sided *P* values presented with a significance threshold of .025.

^c^ Any genitourinary, gastrointestinal, or sexual toxic effect indicates the highest-grade adverse event in that domain for all patients. Patients may have experienced more than 1 category of adverse event.

### Patient-Reported Outcomes

**Figure 3.**
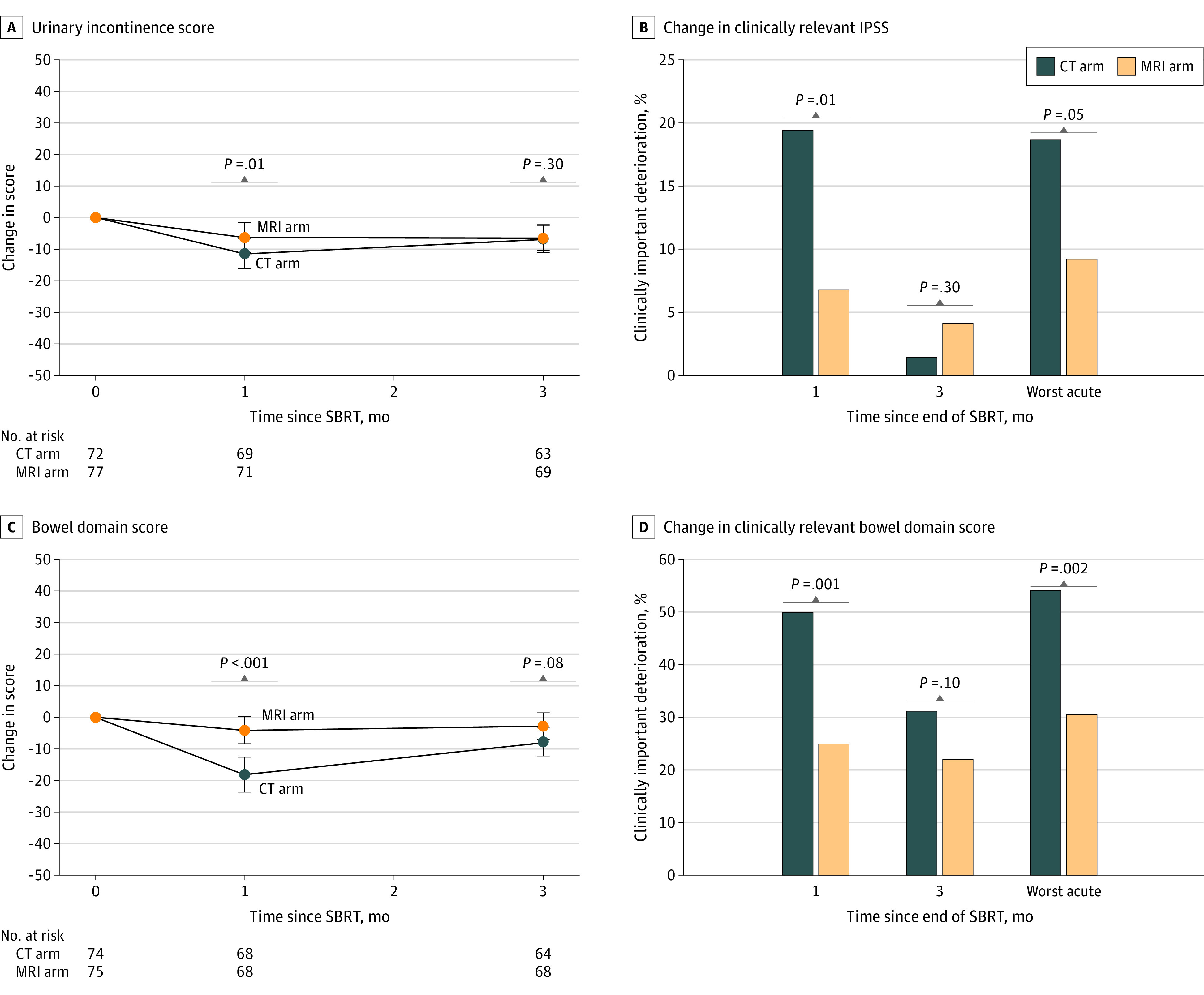
Short-term Changes in Patient-Reported Outcomes A and C, Longitudinal changes in Expanded Prostate Cancer Index Composite-26 (EPIC-26) urinary incontinence and total bowel domain scores. *P* values were determined by the Mann-Whitney test. B and D, Thresholds were 15 or more points (increase) for International Prostate Symptom Score (IPSS) and 12 or more points (decrease) for EPIC-26 bowel domain scores. *P* values were determined by the χ^2^ test. CT indicates computed tomography; MRI, magnetic resonance imaging; SBRT, stereotactic body radiotherapy.

Mean longitudinal changes in urinary and bowel patient-reported outcomes are shown in Figure 3. After SBRT, urinary incontinence subdomain scores decreased significantly more with CT guidance vs MRI guidance at 1 month (11.3-point vs 6.2-point decrement; *P* = .01) but were no longer significantly different at 3 months. No significant differences in urinary-irritative or obstructive subscore or overall urinary domain score were seen (eFigure 1 in Supplement 2). Differences in the percentages of patients reporting a clinically relevant change in urinary incontinence or urinary irritative or obstructive domains did not reach statistical significance at 1 month or 3 months (eFigure 2 in Supplement 2). The percentage of patients with a clinically relevant increase in IPSS (>15 points) was significantly greater with CT guidance at 1 month (19.4% [14 of 74] vs 6.8% [5 of 72]; *P* = .01) but not at 3 months (1.4% [1 of 72] vs 4.1% [3 of 74]; *P* = .30). Differences in total IPSS and IPSS quality of life subscore did not significantly differ (eFigures 4 and 5 in Supplement 2). Magnetic resonance imaging guidance vs CT guidance also resulted in a significantly smaller decrement in EPIC-26 bowel domain subscores at 1 month (4.1-point vs 18.2-point decrement; *P* < .001) but not at 3 months. A significantly larger percentage of patients treated with CT guidance experienced a clinically relevant (≥12-point) decrement in EPIC-26 bowel domain scores (50.0% [34 of 63] vs 25.0% [17 of 63]; *P* = .001). A numerically greater decrement in EPIC-26 sexual domain scores was seen with CT guidance vs MRI guidance at 3 months (13.2-point vs 3-point decrement; *P* = .04; analysis restricted to men who did not receive androgen-deprivation therapy). No differences in longitudinal hormonal or vitality domain score or proportions of patients with clinically relevant decrements in the sexual domain and the hormonal domain were seen (analysis restricted to men who did not receive androgen-deprivation therapy) (eFigures 3 and 4 in Supplement 2).

## Discussion

This randomized clinical trial demonstrated that MRI guidance led to a significant reduction in both physician-scored toxic effects and patient-reported symptom burden in the acute time frame following prostate SBRT. Specifically, acute grade 2 or greater GU and GI toxic effects were reduced with absolute differences of 19.0% for GU and 10.5% for GI, with no grade 3 GU or grade 2 or greater GI toxic events seen in the MRI arm. The percentages of patients experiencing a clinically significant decrease in EPIC-26 urinary incontinence and a large increase in IPSS were significantly lower with MRI guidance at the 1-month time point, as was the percentage of patients with a clinically significant decrement in EPIC-26 bowel scores. The toxicity benefits were likely directly attributable to the significantly reduced PTV margins, which in turn were achievable due to the ability of MRI guidance to frequently monitor intrafraction motion and initiate an automatic beam-hold, as well as to reduced uncertainty in contouring due to a direct MRI-MRI fusion. The benefit of MRI guidance persisted on a multivariable analysis adjusted for other important covariates. Whether the robust differences in short-term toxic effects will persist in the longer term will require additional follow-up. In addition, no differences in clinically relevant EPIC-26 overall urinary scores or in the IPSS quality of life subscore were seen, which should be considered when interpreting the large decrease in physician-scored moderate or greater urinary toxic effects.

The MIRAGE trial was designed using an estimate for toxic effects after CT-guided SBRT that was derived from the Prostate Advances in Comparative Evidence (PACE-B) trial,^1^ which to our knowledge, had published the highest-quality data available for CT-guided SBRT (incidence of grade ≥2 GU toxic effects of 29.2%). The rate of toxic effects in the CT-guided arm in the present trial was higher at 43.4%, which is likely reflective of the higher prescription dose used in the present study (40 Gy to the PTV vs 36.25 Gy in PACE-B). In fact, compared with other prospective (largely nonrandomized) SBRT series, the CT arm of MIRAGE demonstrated the highest rate of acute grade 2 or greater GU toxic effects and the second-highest rate of grade 2 or greater GI toxic effects (with the latter still being less than that in the PACE-B trial) (eTable 7 in Supplement 2). This higher rate of toxic effects can largely be attributed to the high doses used in the MIRAGE trial, as dose escalation has been consistently associated with increased GU and GI toxic effects in the context of prostate radiotherapy.^31,32^ A 40-Gy prescription dose was chosen based on reports suggesting improved biochemical and local control with dose escalation and was justified by the fact that only 19% of patients treated had favorable intermediate-risk disease.^33,34^ Planning target volume margins in the anterior and superior dimensions, which would be most likely to be associated with GU toxic effects, were similar in both trials (4 mm for all patients in MIRAGE and 4-5 mm in 88.2% of patients in PACE-B). Of note, the incidence of grade 2 or greater GI toxic effects was similar, if not lower, in the CT arm of the present study (10.5%) compared with that in PACE-B (15.3%), even though posterior PTV margins less than 4 mm were allowed in the PACE-B trial.^1^ The margins and imaging practices used in the CT arm of MIRAGE were also consistent with those used in the contemporary Stereotactic Body Radiation Therapy or Intensity-Modulated Radiation Therapy in Treating Patients With Stage IIA-B Prostate Cancer (NRG GU-005) trial,^35^ which allowed a minimum PTV margin of 3 mm in the antero-posterior axis and 5 mm in all other directions and did not require intrafraction monitoring more frequently than once every 8 minutes. In contrast, the 2-mm margin used with MRI guidance in MIRAGE was narrower than what, to our knowledge, has been used in any large study. The attributes of the MRI-guided system that allowed us to confidently reduce the margin to 2 mm included improved patient setup (superb soft-tissue contrast of on-board MRI), elimination of uncertainties introduced by MRI-CT fusions when contouring, and real-time cine with extremely rapid intrafraction motion monitoring (4 times per second) including an automatic beam-hold.

It is possible that requiring real-time x-ray–based or electromagnetic-based intrafraction monitoring might have allowed the use of a smaller margin (eg, 2-3 mm isotropically) than was used in the CT arm of the present study. However, it is notable that, to our knowledge, no other prospective series of SBRT has reported no acute grade 2 or greater GI toxic effects (eTable 7 in Supplement 2). Regardless, these forms of image guidance would require an invasive procedure to place intraprostatic markers and potential x-ray exposure to quantify and track motion, which are not risk free.^36,37^ Furthermore, ultimately, these methods rely on tracking an indirect marker of prostate motion rather than prostate motion itself. In addition, the frequency of real-time intrafraction monitoring can be variable; for instance, robotic LINAC systems can image as frequently as once per 30 to 60 seconds, which is still 120 to 240 times less frequent than the MRI-guided system used in this study. The extremely high frequency of intrafraction motion monitoring with an automatic beam-hold as well as improved alignment at the time of treatment initiation may also explain the reduction in GI toxic effects that was seen even though rectal dosimetry did not differ between arms. The lack of dosimetric differences despite the margin difference was likely related to higher conformity with volumetric-modulated arc radiotherapy available in the CT guidance arm compared with step-and-shoot, intensity-modulated radiotherapy in the MRI guidance arm.

Of note, concerns regarding MRI guidance in general pertain largely to logistical issues involving up-front equipment cost and treatment times.^20^ The postimaging delivery time was greater in patients receiving MRI guidance than in those receiving CT guidance (median, 1133 seconds vs 232 seconds). These logistical concerns must be weighed against the benefit to the patients since acute toxic effects from treatment were substantially reduced with MRI guidance. Because adaptive radiotherapy was not performed in this study, the estimated cost differential for the MRI-guided workflow was approximately $1500.^38^

### Limitations

We recognize the limitations inherent to this study. First, blinding was not done, which might have led to attribution bias for physician-scored toxic effects. While use of an independent physician reviewer to adjudicate toxic effects for each patient may have obviated this bias, use of the reviewer would have been impractical for 2 reasons. First, there were obvious differences in the treatment course that patients may have verbalized even if the independent evaluator was not aware of the treatment platform, such as being treated inside a tube (ie, MRI), noting a long treatment time (ie, MRI-LINAC treatments are substantially longer), or commenting on a procedure to place markers in the prostate (ie, fiducial marker insertion, done only in the CT-guided arm). Second, the trial was designed and launched at the height of the COVID-19 pandemic (2 patients died of COVID-19), and auxiliary patient-physician encounters would have been impractical (if even permissible). A second limitation is the single-center setting, which might impact the generalizability of the results. Third, the clinical significance of acute toxicity end points is debatable. Given the overall low cumulative incidence of late toxic effects following modern prostate SBRT, a randomized clinical trial to detect a difference in late toxic effects would have been prohibitively large in the single-center setting. Although MRI-guided LINACs are available at a variety of centers, it is notable that MIRAGE is, to date, the only randomized clinical trial of MRI guidance vs CT guidance in this setting, with no other studies planned, to our knowledge. Moreover, it should be noted that it is implausible that late toxic effects would be worse with MRI guidance. Nonetheless, we will continue to monitor toxicity outcomes, and an analysis of 2-year patient-reported outcomes is planned. Fourth, while certain patient-reported urinary metrics were improved with MRI guidance, no differences in clinically relevant decrement in total urinary score was seen, raising the question of the clinical relevance of the large decrease in physician-scored toxic effects seen with MRI guidance. Fifth, sexual dysfunction is a known sequela of all types of radiation, including SBRT. With only 90 days of follow-up and 68% of patients receiving hormonal therapy, a rigorous comparison of sexual outcomes between arms would be neither possible nor informative at this early time point; however, a longer-term analysis of sexual outcomes is planned. Despite the limited sample size for analysis, a numerical difference in the longitudinal change in sexual quality of life scores favoring the MRI guidance arm was noted at 3 months.

## Conclusions

In this, to our knowledge, first phase 3 randomized clinical trial comparing CT-guided SBRT with MRI-guided SBRT for localized prostate cancer, our results demonstrated that the aggressive margin reduction afforded by MRI guidance allowed a substantial reduction in acute physician-scored toxic effects as well as multiple patient-reported outcome metrics. Longer-term follow-up is necessary to determine whether differences in late urinary or bowel toxic effects will occur and to evaluate differences in sexual outcomes.

## Notes

Supplement 1.
Trial Protocol

Supplement 2. **eTable 1.** Details on Treatment Delivery Times **eTable 2.** Comparison of Dosimetric Parameters Between CT- and MRI-Guided Arms **eTable 3.** Sensitivity Analysis for Differences in Acute Genitourinary and Gastrointestinal Toxicity Including Patients Not Who Were Analyzed But Not Evaluable **eTable 4.** Multivariate Analysis for Acute Grade ≥2 Genitourinary Toxicities **eTable 5.** Differences in Grade ≥2 Genitourinary and Gastrointestinal Toxicities Stratified by Baseline International Prostate Symptom Score (IPSS) **eTable 6.** Differences in Grade ≥2 Genitourinary and Gastrointestinal Toxicities Stratified by Baseline Prostate Volume **eTable 7.** Acute Grade ≥2 Genitourinary and Gastrointestinal Toxicity Rates in Selected Other Stereotactic Body Radiotherapy Studies **eFigure 1.** Longitudinal Changes in Urinary Irritative/Obstructive and Total Urinary Expanded Prostate Cancer Index Composite-26 (EPIC-26) Scores **eFigure 2.** Proportions of Patients With Clinically Relevant Declines in Expanded Prostate Cancer Index Composite-26 (EPIC-26) Scores Urinary Subdomains **eFigure 3.** Longitudinal Changes in Total International Prostate Symptom Score (IPSS) and IPSS Quality-of-Life Scores **eFigure 4.** Longitudinal Changes in Sexual and Hormonal Expanded Prostate Cancer Index Composite (EPIC-26) Scores **eFigure 5.** Proportions of Patients With Clinically Relevant Declines in Sexual and Hormonal Expanded Prostate Cancer Index Composite (EPIC-26) Scores
eReferences

Supplement 3.
Data Sharing Statement

## References

[1] Brand DH, Tree AC, Ostler P, ; PACE Trial Investigators. Intensity-modulated fractionated radiotherapy versus stereotactic body radiotherapy for prostate cancer (PACE-B): acute toxicity findings from an international, randomised, open-label, phase 3, non-inferiority trial. Lancet Oncol. 2019;20(11):1531-1543. doi:10.1016/S1470-2045(19)30569-8 31540791 PMC6838670

[2] Widmark A, Gunnlaugsson A, Beckman L, . Ultra-hypofractionated versus conventionally fractionated radiotherapy for prostate cancer: 5-year outcomes of the HYPO-RT-PC randomised, non-inferiority, phase 3 trial. Lancet. 2019;394(10196):385-395. doi:10.1016/S0140-6736(19)31131-6 31227373

[3] van Dams R, Jiang NY, Fuller DB, . Stereotactic Body Radiotherapy for High-Risk Localized Carcinoma of the Prostate (SHARP) Consortium: analysis of 344 prospectively treated patients. Int J Radiat Oncol Biol Phys. 2021;110(3):731-737. doi:10.1016/j.ijrobp.2021.01.016 33493615 PMC8956505

[4] Kishan AU, Dang A, Katz AJ, . Long-term outcomes of stereotactic body radiotherapy for low-risk and intermediate-risk prostate cancer. JAMA Netw Open. 2019;2(2):e188006. doi:10.1001/jamanetworkopen.2018.8006 30735235 PMC6484596

[5] Schaeffer E, Srinivas S, Antonarakis ES, . NCCN Guidelines insights: prostate cancer, version 1.2021. J Natl Compr Canc Netw. 2021;19(2):134-143. doi:10.6004/jnccn.2021.0008 33545689

[6] Soerjomataram I, Lortet-Tieulent J, Parkin DM, . Global burden of cancer in 2008: a systematic analysis of disability-adjusted life-years in 12 world regions. Lancet. 2012;380(9856):1840-1850. doi:10.1016/S0140-6736(12)60919-2 23079588

[7] Donovan JL, Hamdy FC, Lane JA, ; ProtecT Study Group. Patient-reported outcomes after monitoring, surgery, or radiotherapy for prostate cancer. N Engl J Med. 2016;375(15):1425-1437. doi:10.1056/NEJMoa1606221 27626365 PMC5134995

[8] Hoffman KE, Penson DF, Zhao Z, . Patient-reported outcomes through 5 years for active surveillance, surgery, brachytherapy, or external beam radiation with or without androgen deprivation therapy for localized prostate cancer. JAMA. 2020;323(2):149-163. doi:10.1001/jama.2019.20675 31935027 PMC6990712

[9] Eton DT, Lepore SJ. Prostate cancer and health-related quality of life: a review of the literature. Psychooncology. 2002;11(4):307-326. doi:10.1002/pon.572 12203744 PMC2593110

[10] Hall WA, Paulson E, Li XA, . Magnetic resonance linear accelerator technology and adaptive radiation therapy: an overview for clinicians. CA Cancer J Clin. 2022;72(1):34-56. doi:10.3322/caac.21707 34792808 PMC8985054

[11] Pathmanathan AU, van As NJ, Kerkmeijer LGW, . Magnetic resonance imaging-guided adaptive radiation therapy: a “game changer” for prostate treatment? Int J Radiat Oncol Biol Phys. 2018;100(2):361-373. doi:10.1016/j.ijrobp.2017.10.020 29353654

[12] Persson E, Emin S, Scherman J, . Investigation of the clinical inter-observer bias in prostate fiducial marker image registration between CT and MR images. Radiat Oncol. 2021;16(1):150. doi:10.1186/s13014-021-01865-8 34399806 PMC8365967

[13] Pathmanathan AU, Schmidt MA, Brand DH, Kousi E, van As NJ, Tree AC. Improving fiducial and prostate capsule visualization for radiotherapy planning using MRI. J Appl Clin Med Phys. 2019;20(3):27-36. doi:10.1002/acm2.12529 30756456 PMC6414142

[14] Antolak JA, Rosen II. Planning target volumes for radiotherapy: how much margin is needed? Int J Radiat Oncol Biol Phys. 1999;44(5):1165-1170. doi:10.1016/S0360-3016(99)00117-0 10421551

[15] Willigenburg T, van der Velden JM, Zachiu C, . Accumulated bladder wall dose is correlated with patient-reported acute urinary toxicity in prostate cancer patients treated with stereotactic, daily adaptive MR-guided radiotherapy. Radiother Oncol. 2022;171:182-188. doi:10.1016/j.radonc.2022.04.022 35489444

[16] Mylona E, Acosta O, Lizee T, . Voxel-based analysis for identification of urethrovesical subregions predicting urinary toxicity after prostate cancer radiation therapy. Int J Radiat Oncol Biol Phys. 2019;104(2):343-354. doi:10.1016/j.ijrobp.2019.01.088 30716523

[17] Alayed Y, Davidson M, Quon H, . Dosimetric predictors of toxicity and quality of life following prostate stereotactic ablative radiotherapy. Radiother Oncol. 2020;144:135-140. doi:10.1016/j.radonc.2019.11.017 31809979

[18] Spratt DE, Lee JY, Dess RT, . Vessel-sparing radiotherapy for localized prostate cancer to preserve erectile function: a single-arm phase 2 trial. Eur Urol. 2017;72(4):617-624. doi:10.1016/j.eururo.2017.02.007 28233591

[19] Keall PJ, Brighi C, Glide-Hurst C, . Integrated MRI-guided radiotherapy—opportunities and challenges. Nat Rev Clin Oncol. 2022;19(7):458-470. doi:10.1038/s41571-022-00631-3 35440773

[20] Tocco BR, Kishan AU, Ma TM, Kerkmeijer LGW, Tree AC. MR-guided radiotherapy for prostate cancer. Front Oncol. 2020;10:616291. doi:10.3389/fonc.2020.616291 33363041 PMC7757637

[21] Ma TM, Lamb JM, Casado M, . Magnetic resonance imaging-guided stereotactic body radiotherapy for prostate cancer (MIRAGE): a phase III randomized trial. BMC Cancer. 2021;21(1):538. doi:10.1186/s12885-021-08281-x 33975579 PMC8114498

[22] Barry MJ, Fowler FJ Jr, O’Leary MP, ; The Measurement Committee of the American Urological Association. The American Urological Association symptom index for benign prostatic hyperplasia. J Urol. 1992;148(5):1549-1557. doi:10.1016/S0022-5347(17)36966-5 1279218

[23] Klüter S. Technical design and concept of a 0.35 T MR-Linac. Clin Transl Radiat Oncol. 2019;18:98-101. doi:10.1016/j.ctro.2019.04.007 31341983 PMC6630153

[24] Pham J, Savjani RR, Gao Y, . Evaluation of T2-weighted MRI for visualization and sparing of urethra with MR-guided radiation therapy (MRgRT) on-board MRI. Cancers (Basel). 2021;13(14):3564. doi:10.3390/cancers13143564 34298777 PMC8307202

[25] Levin-Epstein R, Qiao-Guan G, Juarez JE, . Clinical assessment of prostate displacement and planning target volume margins for stereotactic body radiotherapy of prostate cancer. Front Oncol. 2020;10:539. doi:10.3389/fonc.2020.00539 32373529 PMC7177009

[26] Green OL, Rankine LJ, Cai B, . First clinical implementation of real-time, real anatomy tracking and radiation beam control. Med Phys. 2018. doi:10.1002/mp.13002 29807390

[27] US Department of Health and Human Services, National Institutes of Health, National Cancer Institute. Common Terminology Criteria for Adverse Events (CTCAE), version 4.03. June 14, 2010. Accessed November 15, 2022. https://www.eortc.be/services/doc/ctc/ctcae_4.03_2010-06-14_quickreference_5x7.pdf

[28] Szymanski KM, Wei JT, Dunn RL, Sanda MG. Development and validation of an abbreviated version of the Expanded Prostate Cancer Index Composite instrument for measuring health-related quality of life among prostate cancer survivors. Urology. 2010;76(5):1245-1250. doi:10.1016/j.urology.2010.01.027 20350762 PMC3152317

[29] Skolarus TA, Dunn RL, Sanda MG, ; PROSTQA Consortium. Minimally important difference for the Expanded Prostate Cancer Index Composite short form. Urology. 2015;85(1):101-105. doi:10.1016/j.urology.2014.08.044 25530370 PMC4274392

[30] Bruynzeel AME, Tetar SU, Oei SS, . A prospective single-arm phase 2 study of stereotactic magnetic resonance guided adaptive radiation therapy for prostate cancer: early toxicity results. Int J Radiat Oncol Biol Phys. 2019;105(5):1086-1094. doi:10.1016/j.ijrobp.2019.08.007 31419510

[31] Michalski JM, Moughan J, Purdy J, et al. Effect of standard vs dose-escalated radiation therapy for patients with intermediate-risk prostate cancer: the NRG Oncology RTOG 0126 randomized clinical trial. *JAMA Oncol*. 2018;4(6):e180039. doi:10.1001/jamaoncol.2018.0039 29543933 PMC5885160

[32] Rodda S, Tyldesley S, Morris WJ, et al. ASCENDE-RT: an analysis of treatment-related morbidity for a randomized trial comparing a low-dose-rate brachytherapy boost with a dose-escalated external beam boost for high- and intermediate-risk prostate cancer. Int J Radiat Oncol Biol Phys. 2017;98(2):286-295. doi:10.1016/j.ijrobp.2017.01.008 28433432

[33] Zelefsky MJ, Kollmeier M, McBride S, . Five-year outcomes of a phase 1 dose-escalation study using stereotactic body radiosurgery for patients with low-risk and intermediate-risk prostate cancer. Int J Radiat Oncol Biol Phys. 2019;104(1):42-49. doi:10.1016/j.ijrobp.2018.12.045 30611838 PMC7525798

[34] Levin-Epstein RG, Jiang NY, Wang X, . Dose-response with stereotactic body radiotherapy for prostate cancer: a multi-institutional analysis of prostate-specific antigen kinetics and biochemical control. Radiother Oncol. 2021;154:207-213. doi:10.1016/j.radonc.2020.09.053 33035622 PMC7956167

[35] Stereotactic body radiation therapy or intensity-modulated radiation therapy in treating patients with stage IIA-B prostate cancer. ClinicalTrials.gov identifier: NCT03367702. Updated August 30, 2022. Accessed November 15, 2022. https://clinicaltrials.gov/ct2/show/NCT03367702

[36] de Crevoisier R, Bayar MA, Pommier P, . Daily versus weekly prostate cancer image guided radiation therapy: phase 3 multicenter randomized trial. Int J Radiat Oncol Biol Phys. 2018;102(5):1420-1429. doi:10.1016/j.ijrobp.2018.07.2006 30071296

[37] Mendenhall WM, Glassman G, Morris CG, . Bacterial urinary tract infection after transrectal placement of fiducial markers prior to proton radiotherapy for prostate cancer. Int J Part Ther. 2016;3(1):21-26. doi:10.14338/IJPT-16-00007.1 31772972 PMC6871580

[38] Parikh NR, Clark MA, Patel P, . Time-driven activity-based costing of CT-guided vs MR-guided prostate SBRT. Appl Radiat Oncol. 2021;10(3):33-40. 34671700 PMC8525878

