# Supplementary material for: Magnetic Resonance Imaging–Guided vs Computed Tomography–Guided Stereotactic Body Radiotherapy for Prostate Cancer: The MIRAGE Randomized Clinical Trial
Source: JAMA Oncol. 2023 Jan 12;9(3):365–73. doi: 10.1001/jamaoncol.2022.6558 (PMC9857817; doi:10.1001/jamaoncol.2022.6558)
Supplement: Supplement 1. — Trial Protocol [file jamaoncol-e226558-s001.pdf]

MAGNETIC RESONANCE IMAGING-GUIDED STEREOTACTIC BODY RADIOTHERAPY FOR PROSTATE CANCER (MIRAGE): A PHASE III RANDOMIZED TRIAL

Organizing Study Center:
UCLA Jonsson Comprehensive Cancer Center
Department of Radiation Oncology
200 UCLA Medical Plaza, Suite B-265
Los Angeles, CA 90095
(310) 825-9775

Principal Investigator:
Amar U. Kishan, MD
Assistant Professor
Department of Radiation Oncology

Co- Principal Investigator:
James M. Lamb, PhD
Associate Professor
Department of Radiation Oncology

Co-Investigators:

Michael L. Steinberg, MD
Professor and Chair
Department of Radiation Oncology

Minsong Cao, PhD
Associate Professor
Department of Radiation Oncology

Yingli Yang, PhD
Associate Professor
Department of Radiation Oncology

Nicholas G. Nickols, MD
Assistant Professor
Department of Radiation Oncology

Nzhde Agazaryan, PhD
Professor
Department of Radiation Oncology

Daniel Low, PhD
Professor
Department of Radiation Oncology

Holly Wilhalme, MSc
Principal Biostatistician
Department of Medicine Statistics Core

T. Martin Ma, MD, PhD
Chief Resident
Department of Radiation Oncology

Protocol Overview

Study Design: Randomized Trial
Sample size: 154 patients (NB: based on interim analysis)
Randomization: Arm 1: CT-guided stereotactic body radiotherapy
Arm 2: MRI-guided stereotactic body radiotherapy
Primary Outcome: acute physician-scored genitourinary toxicity
Secondary Outcome: acute physician-scored gastrointestinal toxicity, late physician-scored toxicity, patient-reported quality of life outcomes, 5-year biochemical recurrence-free survival

Treatment Details:

SBRT Prostate	SBRT pelvic nodes (optional)	Hormonal Therapy (optional)	Follow-up Schedule	Tests at each Follow-up
40 Gy in 5 fractions of 8 Gy	25 Gy in 5 fractions of 5 Gy	4-36 months*	q 3 months year 1<br>q 6 months year 2-5<br>q yearly thereafter	PSA<br>CTCAE toxicity scores<br>EPIC-26 scores<br>IPSS scores<br>SHIM scores

Radiation Target Doses:
Prostate±Seminal Vesicles PTV (PTV_P): 40 Gy in 5 fractions, 8 Gy per fraction
Pelvic Nodal PTV (PTV_N): 25 Gy in 5 fractions, 5 Gy per fraction (optional)
Treatments preferably delivered every other day or may be given consecutive days, with treatment within a period of time may not have exceeded 14 days.

RT Technique: MRI-guided radiotherapy or CT-guided radiotherapy

Hormonal Therapy: Delivered per physician discretion, generally in the form of total androgen deprivation therapy for 4 months followed by LHRH or GnRH agonist or antagonist alone for 0-32 months depending on the patient's NCCN risk group.

INDEX

PROTOCOL SYNOPSIS

SCHEMA

1.0 OBJECTIVES AND ENDPOINTS

2.0 BACKGROUND

3.0 PATIENT SELECTION

4.0 REGISTRATION AND RANDOMIZATION PROCESS

5.0 TREATMENT PLAN

6.0 ANDROGEN DEPRIVATION THERAPY

7.0 PATIENT ASSESSMENTS AFTER TREATMENT

8.0 STUDY CALENDAR

9.0 DATA REPORTING AND REGULATORY CONSIDERATIONS

10.0 STATISTICAL CONSIDERATIONS

11.0 COST CONSIDERATIONS

12.0 REFERENCES

APPENDIX: Informed Consent
EPIC-26 short form
IPSS
SHIM

SYNOPSIS

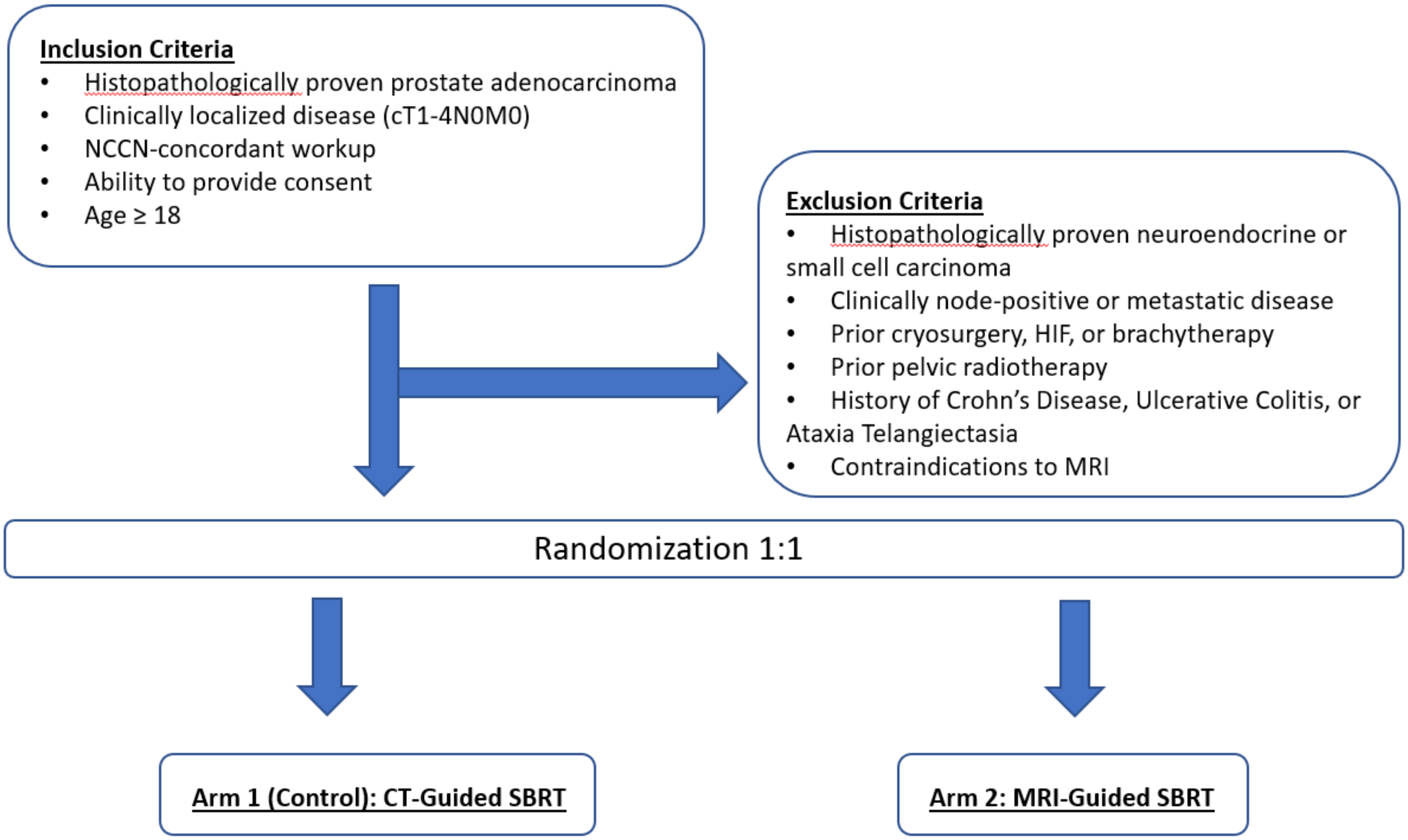
Inclusion Criteria
• Histopathologically proven prostate adenocarcinoma
• Clinically localized disease (cT1-4N0M0)
• NCCN-concordant workup
• Ability to provide consent
• Age ≥ 18
Exclusion Criteria
• Histopathologically proven neuroendocrine or small cell carcinoma
• Clinically node-positive or metastatic disease
• Prior cryosurgery, HIF, or brachytherapy
• Prior pelvic radiotherapy
• History of Crohn's Disease, Ulcerative Colitis, or Ataxia Telangiectasia
• Contraindications to MRI
Randomization 1:1
Arm 1 (Control): CT-Guided SBRT
Arm 2: MRI-Guided SBRT

CLINICAL STUDY SCHEMA

STUDY DESIGN

Randomized trial designed to evaluate the superiority of magnetic resonance imaging (MRI)-guided stereotactic body radiotherapy (SBRT) over standard computed tomography (CT)-guided SBRT for prostate cancer (PCa) with respect to acute physician-scored genitourinary (GU) toxicity. Acute physician-scored gastrointestinal (GI) toxicity, late physician-scored toxicity and patient-reported quality of life, and five-year biochemical recurrence-free survival will also be evaluated.

NUMBER OF PATIENTS

We plan to enroll 154 patients (**NB: this is following an interim analysis that allowed a revision of the power calculation; the original sample size estimate was 300 patients)**. The rate of accrual is expected to be in the range of 100 patients per year.

INTERVENTION AND MODE OF DELIVERY

Standard of care SBRT for localized PCa, delivered either via an MRI-guided linear accelerator or a CT-guided linear accelerator.

DURATION OF INTERVENTION AND EVALUATION

Five fractions of SBRT will be delivered over 14 days, and patients will be followed for 5 years per routine standard of care.

STATISTICAL METHODS/SAMPLE SIZE JUSTIFICATION

The primary objective of this study will be to determine whether MRI-guided SBRT can lead to a 14% absolute reduction in the cumulative incidence of acute physician-scored ≥2 GU toxicity (defined by the Common Terminology Criteria for Adverse Events version 4.03) when compared to a rate of 29% in (CT)-guided SBRT group, corresponding to a relative risk reduction of 52%. As detailed in section 10.2 of the protocol, an overall sample size of 300 patients (n=150 each arm) will provide 83.7% power to detect an absolute risk reduction of 14% in acute physician-scored ≥2 GU toxicity with a one-sided two-sample Z-test at, a 0.025 significance level. The event rate may be higher than anticipated as the doses to be used on the MIRAGE trial are larger than those used on previous prostate SBRT trials; therefore, an interim analysis will be performed after 100 patients are enrolled to revise the estimated sample size. **(NB: The interim analysis suggested a revised sample size estimate of 154 patients).**

FUNDING, REGULATORY, AND FEASIBILITY ISSUES

The treating department will have the capability, equipment, and expertise to perform image-guided SBRT. Androgen deprivation, clinical evaluation and labs, and radiographic follow-up when necessary will be performed according to standard of care.

PATIENT ACCEPTABILTY/ETHICS AND CONSENT ISSUES

Only patients able to give informed consent will be eligible for the study.

1.0 OBJECTIVES AND ENDPOINTS

PRIMARY OBJECTIVE:

1. To determine whether (MRI)-guided stereotactic body radiotherapy (SBRT) improves acute physician-scored genitourinary (GU) toxicity when compared with standard computed tomography (CT)-guided SBRT for prostate cancer (PCa). Acute GU toxicity will be assessed by the Common Terminology Criteria for Adverse Events (CTCAE) version 4.03 scale.

SECONDARY OBJECTIVES:

1. To determine whether there are differences in acute grade ≥2 GI toxicity as assessed by the CTCAE version 4.03 scale, following MRI-guided SBRT versus CT-guided SBRT.

2. To determine whether there are differences in 5-year cumulative incidences of late grade ≥2 GU and GI physician-reported toxicity, following MRI-guided SBRT versus CT-guided SBRT.

3. To quantify the temporal changes in patient-reported quality of life (QOL) outcomes, as assessed by the Expanded Prostate Cancer Index-26 (EPIC-26), International Prostate Symptom Scores (IPSS), and Sexual Health Inventory for Men (SHIM) QOL indices, following MRI-guided SBRT.

4. To determine whether there are differences in 5-year biochemical recurrence-free survival (BCRFS) following MRI-guided SBRT.

PRIMARY ENDPOINT:

1. The incidence of acute grade ≥2 GU physician-reported toxicity, as assessed by the CTCAE version 4.03 scale. The timeframe will be restricted to the first 90 days after SBRT.

SECONDARY ENDPOINTS:

1. The incidence of acute grade ≥2 GI toxicity as assessed by the CTCAE version 4.03 scale. The timeframe will be restricted to the first 90 days after SBRT.

2. The 5-year cumulative incidences of late grade ≥2 GU and GI physician-reported toxicity, as assessed by the CTCAE version 4.03 scale.

3. The temporal changes in patient-reported QOL outcomes will be obtained depending on the instrument used. For the EPIC-26 instrument, these will be represented by changes from baseline in the urinary incontinence, urinary obstruction, bowel, sexual function, and hormone/vitality domains. Changes will be analyzed with respect to whether they represent minimally important differences.[1] For the IPSS and SHIM instruments, the numerical change from baseline, as well as the raw score at any given timepoint, will be extracted.

4. Five-year BCRFS, with biochemical recurrence (BCR) defined as serum PSA levels that are 2 ng/mL higher than the nadir PSA achieved after SBRT.

2.0 BACKGROUND

2.1 The Role of Stereotactic Body Radiotherapy in the Definitive Treatment of Prostate Cancer

Most patients diagnosed with prostate cancer (PCa) in the developed world present with clinically localized disease, and the majority have low-risk or intermediate-risk disease as defined by the National Comprehensive Cancer Network (NCCN).[2] Multiple management options are available, including definitive external beam radiotherapy (RT), radical prostatectomy, brachytherapy, and (for patients with low-risk and favorable intermediate-risk disease) active surveillance. The traditional course of RT, called conventionally fractionated RT (CF-RT) consists of small doses or fractions of radiation (1.8 to 2.0 Gy each) delivered over 39 to 45 treatment sessions. Considerable data suggest that PCa may exhibit an enhanced sensitivity to higher doses per fraction compared to most other tumors and even normal tissues.[3] This has driven considerable research focused on RT regimens that deliver higher doses per fraction. Moderate hypofractionation (MHF-RT), or using fractions of 2.4-3.4 Gy over the span of 20 to 30 treatment session, has been studied extensively, with three noninferiority randomized clinical trials demonstrating the efficacy and safety of this approach[4-6] and one study showing superiority.[7] This is now considered a standard approach for localized PCa.[8]

Extreme or ultrahypofractionation (UF-RT), or using fractions of 5 Gy or higher, is a newer approach that would allow treatment completion in four to seven treatment sessions. When delivered in five or fewer fractions with the aid of sophisticated delivery platforms, this is termed stereotactic body radiotherapy or stereotactic ablative radiotherapy (SBRT/SABR). Based on early efficacy and safety reports,[9] SBRT was first included in the NCCN guidelines in 2014 as a potential option for patients with localized disease. As of 2019, the NCCN guidelines have been revised to include SBRT as a standard of care option for men with low and favorable intermediate-risk disease. [2] Additionally, SBRT is noted as an option for patients with unfavorable intermediate-risk and high-risk disease in the setting of logistical impediments to longer courses of radiotherapy. Three landmark studies, which are briefly reviewed below, were critical to this change in the guidelines.

The landmark HYPO-RT-PC compared 5-year failure-free survival (FFS) between patients with intermediate- and high-risk disease who received 42.7 Gy in seven fractions of 6.1 Gy (delivered over 2.5 weeks) and those who received 79 Gy in 38 fractions of 2 Gy.[10] Overall, 1200 men (89% with intermediate risk disease) were enrolled across 12 Swedish centers between 2005-2015. With a median follow-up time of 5 years, the 5-year FFS rates were 84% in both groups, with an adjusted hazard ratio of 1.002 (95% CI 0·758−1·325; log-rank p=0·99), confirming oncologic non-inferiority. The 5-year cumulative incidence of grade ≥2 GU toxicity and gastrointestinal (GI) toxicities were similar in both arms (18% vs. 17% for GU, and 10% vs. 10% for GI). The 5-year cumulative incidence of grade ≥3 GU and GI toxicity were low across both arms (4.2% vs. 4.7% for GU and 1.7% vs. 1.9% for GI). Erectile function decreased from 70% at baseline to 35% at 5 years in both arms.

Importantly, the radiation planning technique used for 80% of patients was three-dimensional conformal radiotherapy, rather than the more modern intensity modulated radiotherapy (IMRT). The latter may be associated with lower absolute rates of toxicity.[11] Moreover, while 90% of patients did have implanted fiducial markers to help mitigate the impact of prostate motion between fractions, the planning

margins used were 7 mm isotropically, which would be considered large by contemporary standards (i.e., a large volume of tissue was exposed to radiation). Thus, the absolute rates of toxicity in the HYPO-RT-PC (on either arm) are likely higher than what would be expected with modern treatment planning and delivery, and the relative importance of this difference for patients receiving CF-RT and UF-RT is unclear.

The PACE-B trial, directly compared modern SBRT (a regimen of 36.25 Gy in five fractions of 7.25 Gy each delivered consecutively [20.7%] or over the span of generally 2 weeks [79.3%]) with a control arm that allowed either CF-RT (78 Gy in 39 fractions of 2 Gy each; 31% of patients) or MF-RT (62 Gy in 20 fractions of 3.1 Gy each; 69% of patients).[12] This trial, which enrolled 874 men with low- and intermediate-risk PCa across 35 centers in the United Kingdom and Canada between 2012-2018, was designed to demonstrate the non-inferiority of SBRT with respect to freedom from biochemical or clinical failure at five years with a non-inferiority margin of 6%.

The authors recently reported the results of a pre-specified substudy evaluating acute physician-scored toxicity on the RTOG scale. The substudy analysis was designed to exclude a 10% increase in RTOG grade ≥2 GI toxicity (from 25%) and 11% increase in GU toxicity (from 40%) with SBRT. The authors found that the rates of worst RTOG grade ≥2 GI toxicities exceeding baseline were 9.3% vs. 13.2% (SBRT vs. control arm), while for grade ≥2 GU toxicities, the rates were 20.2% vs. 26.8%. These typically occurred 4-6 week after the end of treatment. By 12 weeks, the rates of worst RTOG grade ≥2 GI toxicities exceeding baseline had dropped to 1.7% vs. 0.5%, with GU rates down to 5.0% vs. 3.8%. No significant differences in were identified at any time point. Similarly, no differences were found in the secondary endpoints of EPIC bowel, urinary, and sexual bother, or worse acute grade ≥2 GU toxicity exceeding baseline on the CTCAE scale.

When directly comparing acute grade ≥2 RTOG GU toxicity rates, the rates were lower in PACE-B than HYPO-RT-PC (20.8% vs 28%), potentially reflective of the use of IMRT. However, only 73% of patients treated with SBRT had implanted fiducial markers, and only 41.7% of patients had motion monitoring during treatment. While there is minimal high-level evidence suggesting that more rigorous motion management strategies are needed, it is conceivable that more sophisticated motion management strategies could allow smaller margins around the target, thereby reducing the amount of normal tissue receiving radiation. Nonetheless, margins were tight, with 5 mm isotropically, except 3-5 mm in the posterior direction. Additionally, longer intervals between SBRT fractions have been associated with decreased toxicity in prior studies,[13,14] and an acute increase in physician-reported gastrointestinal (GI) toxicity has been noted with moderate hypofractionation.[8] Daily fractionation was used for 20.7% of patients treated with SBRT on the PACE-B, and 69% of patients treated on the control arm received a moderately hypofractionated regimen. Thus, further reductions in toxicity rates could be seen simply by analyzing data based on fractionation; however, such data have not yet been published.[15]

Finally, a large dataset with longer-term follow-up for SBRT comes from a pooled consortium report of 12 single arm phase II studies that enrolled 2142 patients between 2000-2012.[14] Regimens ranged from 38 Gy in four fractions of 9.5 Gy each to 40 Gy in five fractions of 8 Gy each. The trials generally enrolled patients with low-risk (55.3%) and favorable intermediate-risk disease (32.3%), though a minority had unfavorable intermediate-risk disease (12.4%). The cumulative incidences of late grade ≥3 or higher

toxic events by either RTOG or CTCAE scales (as defined by the individual studies included) were evaluated based on central review. With a median follow-up of 6.9 years, the seven-year cumulative incidence of late grade ≥3 GU toxicities was 2.4%, and the rate of grade ≥3 GU toxicities was 0.4%. The seven-year cumulative incidence biochemical recurrence was 4.5% for low-risk disease, 8.6% for favorable intermediate-risk disease, and 14.9% for unfavorable intermediate-risk disease.

Though not randomized evidence, this report provides prospectively-collected multi-institutional data in a large cohort of patients treated with SBRT. Of note, the patients included in this report received treatments with protocols most reflective of modern SBRT delivery. For instance, all patients had implanted fiducial markers to guide motion management and 88% of men had real-time monitoring of prostate motion during radiation. The majority of the remainder had at least interval imaging to account for prostate motion during treatment, and margins ranged from 2 mm to 5 mm isotropically. Indeed, the low absolute rates of grade ≥3 toxicity seen at seven years (over three-fold less than in the HYPO-RT-PC trial) may be secondary to these technological improvements.

Overall, as of 2019, SBRT is now considered a standard of care option for men with low and favorable intermediate risk prostate cancer and is listed as an acceptable alternative for patients with unfavorable intermediate and high risk prostate cancer who have logistical difficulties with longer courses of radiotherapy. Economic considerations are also worthy of discussion, given the increasing incidence of PCa and the projected $18.53 billion annual cost of PCa care in 2020.[16,17] A recently published time-driven activity-based costing analysis of various treatment modalities for low-risk PCa estimated the average cost of a course of IMRT to be $23,565, versus $8,978 for low-dose rate brachytherapy and $11,665 for SBRT.[18] A Markov decision model also found that the average cost of SBRT was substantially lower than IMRT ($35,431 vs. $22,152), as did a Medicare claims analysis ($13,645 vs. $21,023).[19,20] Future research, then, should be directed at optimizing the therapeutic window of SBRT further.

2.2 The Role of Magnetic Resonance Guided Radiotherapy

As discussed above, there is considerable evidence supporting the safety and efficacy of prostate SBRT. Oncologic efficacy is high and severe toxicity rates in both the acute and late settings are reasonably low. However, the incidences of acute and late grade ≥2 are not inconsequential. The best estimates of grade ≥2 toxicities following modern SBRT come from the PACE-B trial (acute)[12] and a multi-center SBRT trial run in the United States.[21] Overall, one would expect modern SBRT to have acute grade ≥2 GU and GI toxicity rates of 29.1% and 16.0%, and late grade ≥2 GU and GI toxicity rates of 13.3% and 2.0%. Additionally, the safe deliver of SBRT does require the use of on-board imaging, which does result in higher doses of radiation being delivered to patients.[22] Safe SBRT delivery also generally requires the placement of implanted fiducial markers to help mitigate prostatic motion during and between radiotherapy fractions,[23] which is an invasive procedure with a risk, albeit a low one, of complications.

Due to the aforementioned prostate motion--both intrafraction (during fractions) and interfraction motion (between fractions)--margins must be placed around the prostate when targeting it with external radiation in order to assure adequate target dosing. This leads to the irradiation of portions of organs that

are adjacent to the prostate, such as the bladder and rectum; it is known that doses (particularly high doses delivered to small areas) are primary drivers of GU and GI toxicity after radiation are doses deposited.[24] In addition to uncertainties due to target motion, additional margins must be placed around the prostate target because of geometric uncertainty and patient setup errors. The prostate is seen more clearly on MRI than on CT-based imaging, such that prostate target volumes generated by MRI are smaller and more reproducible than those generated by CT.[25] While MRIs can be used to help generate CT-based contours through image fusion, this fusion process itself introduces 1-2 mm of residual error.[26] Treatment on an MRI-based device, which has significantly improved soft-tissue resolution, would allow more accurate target delineation by bypassing the need for CT-MRI fusion. The MRI-guided linear accelerator (LINAC) system being evaluated in this protocol (MRIdian System™, ViewRay™, Cleveland, OH, USA) has the capability of performing real-time imaging via the use of cine MRI imaging. This will allow the monitoring of prostate motion in real-time, abrogating the need for implanted fiducials and eliminating the need for frequent X-ray based imaging to detect these fiducials. It will also allow for narrower margins around the prostate target because the boundaries between the prostate and nearby important healthy tissues such as the bladder and rectum can be precisely visualized with on-board MRI. Finally, several critical adjacent structures, such as the bladder, rectum, and bowel, are highly deformable and, despite rigorous patient preparation instructions, will lead to anatomic variability from fraction to fraction. Such deformation cannot be corrected using fiducial marker-based translational and rotational corrections on a conventional linear accelerator with x-ray image guidance. The MRIdian system can uniquely correct for these deformations using online adaptive radiotherapy, wherein a new radiotherapy plan is generated on the basis of the anatomy seen at a given fraction.[27]

Recently, Bruynzeel et al. reported the results of a prospective phase II study of MRI-guided SBRT in 101 with localized prostate cancer (NCT03961321).[28] MRgRT was delivered in 5 fractions of 7.25 Gy to the target volume using daily plan adaptation with simultaneous relative sparing of the urethra to a dose of 6.5 Gy per fraction. Acute CTCAE version 4.0 grade ≥2 GU toxicity incidence was 19.8% at the end of MRI-guided SBRT, while acute CTCAE version 4.0 grade ≥2 GI toxicity was 3.0% at the end of MRI-guided SBRT. These correspond favorably to the rates of 29.1% and 16.0% in PACE-B.[12]

3.0 PATIENT SELECTION

3.1 Inclusion criteria:

3.1.1. Histologically confirmed, clinical localized adenocarcinoma of the prostate

3.1.2. No evidence of disease beyond the prostate and/or seminal vesicles (i.e., no suspicious pelvic lymph nodes or presence of metastatic disease outside the pelvis)

3.1.3. Staging workup as recommended by the NCCN on the basis of risk grouping:

(a) Low risk: No staging workup required

(b) Favorable intermediate-risk: CT abdomen/pelvis if MSKCC nomogram predicts >10% probability of lymph node involvement

(c) Unfavorable intermediate-risk: technetium bone scan, CT abdomen/pelvis if MSKCC nomogram predicts >10% probability of lymph node involvement

(d) High-risk: technetium bone scan, CT abdomen/pelvis if MSKCC nomogram predicts >10% probability of lymph node involvement

(e) Advanced imaging studies (i.e. PSMA PET and Axumin scan) can supplant a bone scan if performed first.

3.1.4. Age ≥ 18

3.1.5. Ability to understand, and willingness to sign, the written informed consent

3.2. Exclusion criteria:

3.2.1. Patients with neuroendocrine or small cell carcinoma of the prostate

3.2.2. Patients with any evidence of distant metastases. Note, evidence of lymphadenopathy below the level of the renal arteries can be deemed loco regional per the discretion of the investigator.

3.2.3. Prior whole gland cryosurgery, HIFU or brachytherapy of the prostate

3.2.4. Prior pelvic radiotherapy

3.2.5. History of Crohn's Disease, Ulcerative Colitis, or Ataxia Telangiectasia

3.2.6. Contraindications to MRI, including: (a) electronic devices such as pacemakers, defibrillators, deep brain stimulators, cochlear implants; (b) metallic foreign body in the eye or aneurysm clips in the brain; (c) severe claustrophobia

4.0 REGISTRATION PROCESS AND RANDOMIZATION

4.1. General guidelines

Patients seen as new patients in consultation in the radiation oncology clinic who are being evaluated for potential definitive radiotherapy for prostate cancer options will be informed of this clinical study if eligible. IRB approved patient flyers, website content, and letters to referring physicians will be used to educate potential patients and referring physicians regarding the study goals and logistics in order to recruit interested patients who have elected to receive SBRT treatment following their prostatectomy. The decision to participate will be entirely voluntary.

4.2. Registration Process

Informed consent form will be given to the patient for review. Consent will be obtained after a clear and thorough discussion between the patient and the treating physician. Whenever feasible and in the best interest of the subject, the informed consent discussion and consent form signing may occur via telemedicine. To register a patient, the research coordinator will obtain or complete: (1) pathological documentation of adenocarcinoma of the prostate (UCLA pathology review not required); (2) signed informed consent form; (3) signed HIPAA authorization form.

Upon confirmation of eligibility and enrollment in the study, the following will be obtained: (1) Medical history, clinical examination and consultation with Radiation Oncology; (2) Signed informed consent. Whenever feasible and in the best interest of the subject, the clinical examination and consultation may occur via telemedicine.

4.3. Randomization

The University of California, Los Angeles (UCLA) Department of Medicine Statistics Core (DOMStat) has built code to randomly assign patients to the two arms after the patient eligibility form is filled out in the Research Electronic Data Capture (REDCap) system. DOMStat has developed a reproducible code to randomly generate the allocation sequence. To ensure balance between treatment allocation throughout the study, a blocked randomization of size 6 will be used. This block size will be blinded to the radiation oncology research team when enrolling a patient. The arm allocation will be masked until after the screening/baseline data are entered and filled out in REDCap (no anticipation of the group assignment will be possible). In order to obtain adequate “allocation concealment”, a list of random allocations has been created for patients 1 through 300. This list is stored in RedCap and will not be modified.

Randomization will be stratified by the baseline International Prostate Symptom Score (IPSS) (≤15 or >15) and prostate gland volume (≤50 cc or >50 cc). Prostate gland volume can be obtained from an MRI, ultrasound, or physical exam, with MRI preferred. For analysis of the secondary endpoints of acute and late physician-scored toxicity, as well as bowel-related patient-reported outcomes, the analysis will be further stratified by the use of hydrogel spacers (use of spacer vs. no spacer used). We will also perform a

post-hoc analysis based on delivery of a simultaneous integrated boost and radiation to the elective lymph node areas. Within each stratum, participants will be assigned in 1:1 ratio within each randomization block to one of the treatment arms:

1. Arm 1 (n=150): The patient will undergo CT-guided SBRT.
2. Arm 2 (n=150): The patient will undergo MRI-guides SBRT.

All the data management will be performed in the online clinical trial database. This is an open label study. Trial participants, care providers, outcome assessors, and data analysts will be aware of the assignment after enrollment is completed. The randomization number and assignment will be communicated by phone or email to the treating physician. Patients will be informed by phone or email of the randomization assignment.

5.0 STUDY TREATMENTS

5.1. Radiotherapy Treatment Platform

Enrolled patients will be treated on with CT-guided SBRT on a standard, gantry-mounted linear accelerator (LINAC) (Arm 1) or with MRI-guided SBRT (Arm 2) on an MRI-guided LINAC (MRIdian System™, ViewRay™, Cleveland, OH, USA).

5.2. Radiotherapy Simulation

Enrolled patients will undergo CT simulation and planning as per routine for patients. Patients will be given a bladder filling protocol with instructions to void one hour prior to simulation (and prior to each treatment), and drink at least two eight ounce glasses of water. This protocol has been shown to stabilize bladder anatomy during SBRT courses.[29] Patients will also be asked to perform a Fleets enema the night before and morning of their simulation scan. At the time of simulation, a custom vacloc bag, alpha cradle, or equivalent immobilization device will be used for patient immobilization and establishment of treatment geometry. A pelvic CT without contrast will be performed for radiotherapy simulation (i.e., treatment planning CT) with a slice thickness of 1.5 mm. For patients enrolled on the MRI-guided SBRT arm, an additional 0.35T MRI will be obtained in the treatment position on the MRI-guided linear accelerator. The predominant sequence will be a True Fast Imaging with Steady State Precession (TRUFI) sequence. Additional sequences, such as HASTE sequences, will be obtained using the onboard 0.35T MRI to assist in urethral delineation. For all patients, a diagnostic strength (1.5-3T) MRI will be fused to the primary planning scan (either a CT or a 0.35T TRUFI MRI) to assist in contouring. The primary fused sequence will be a T2-weighted image. These procedures are all considered standard-of-care for prostate radiotherapy planning.

Implanted fiducial markers are routinely used to assist with motion management when treating prostate cancer patients with any form of external radiotherapy, including SBRT.[30] These will be considered required for patients treated with CT-guided SBRT, as is consistent with our internal SBRT protocol. For patients being treated with MRI-guided SBRT, since the prostate can be visualized with the real-time cine feature, implanted fiducial markers will not be required.

The insertion of a hydrogel spacer between the rectum and the prostate has demonstrated improvements in various metrics in one randomized trial,[31] and it remains unknown whether the use of such spacers would have a proportionately greater effect for patients treated with SBRT. The use of a hydrogel is not an exclusion criteria for the trial, but is not considered mandatory either.

5.3. Radiotherapy Contouring and Planning

The entire prostate will be contoured as the CTV_P. For patients with intermediate and high-risk disease, the proximal 1 cm of seminal vesicles will be included in the CTV_P. For patients with cT3a disease (i.e.., extracapsular disease), the CTV_P will be expanded to cover the radiographic extent of ECE. For patients with cT3b disease, the CTV_P will be expanded to cover the entire seminal vesicle. The CTV_P will be expanded by 2 or 4 mm isotropically depending on randomization to form the PTV_P. For patients

receiving CT-guided SBRT, the PTV expansion will be 4 mm. For those receiving MRI-guided SBRT the expansion will be 2 mm. For selected patients with high-risk disease, whole pelvic radiotherapy (WPRT) can be considered. If WPRT is to be delivered, the obturator, presacral, internal iliac, and external iliac nodal stations will be considered as targets. The nodal CTV (CTV_N) will be defined as per the RTOG consensus guidelines[32]. CTV_N will be expanded isotropically by 4 mm to form PTV_n.

Prescription doses for the various PTVs are shown below. For all PTVs, the prescription dose will be prescribed such that 95% of the PTV receives at least the prescription dose, unless doing so would lead to violation of the organ-at-risk dose constraints listed in section 5.4. In such cases, undercoverage of either the PTV_P or the PTV_N will be allowed per physician discretion.

Table 2. Target Prescription Doses

PTV_{P*}	40 Gy in 5 fractions, 8.0 Gy per fraction
PTV_N	25 Gy in 5 fractions, 5.0 Gy per fraction

*A simultaneous integrated boost to a gross tumor volume (GTV) as defined by a diagnostic MRI and/or advanced nuclear imaging scan can be delivered, up to a maximum dose of 42 Gy in 5 fractions, per investigator discretion

5.4. Organs at Risk (OAR) Dose Constraints

Delineation of normal structures will include the bladder, rectum, rectal wall (anterior and posterior at level of the prostate gland), femoral heads, small bowel, bladder, urethra, skin, and penile bulb. The radiation physicist will optimize the radiation therapy treatment plan and the responsible study investigator will review it prior to approval for treatment. Dose volume histograms (DVH), and normal tissue constraint parameters specified below will be used to judge the quality of the plan and optimize doses to the PTV as well as maximally sparing of OARs.

The dose constraints below for OARs will be used to assess dosimetry. Doses that exceed the constraints below will be considered deviations from the protocol and can be delivered if study investigators agree that the deviation is acceptable and unlikely to cause excessive morbidity. Doses listed are as total over 5 fractions and per fraction. In order to verify each of these limits, the organs must be contoured such that appropriate dose volume histograms can be generated.

Table 3. Organ-At-Risk Dose Constraints

Organ	Volume	Dose (cGy)
Rectum	50% of total volume	20 Gy
	50% of circumference on any given slice	24 Gy

	20% of total volume	32 Gy
	10% of total volume	36 Gy
	5% of total volume	40 Gy
	Anterior rectal wall Maximum point dose*	42 Gy (8.4 Gy per fraction)
	Posterior rectal wall Maximum point dose	16 Gy (3.2 Gy per fraction)
	Highest dose received by 2 cc	38 Gy
Anal Canal (rectum inferior to any PTV)	Maximum point dose	30 Gy (6.0 Gy per fraction)
	Less than 5 cc	20 Gy (4.0 Gy per fraction)
Small intestine	Maximum point dose	25 Gy (5.0 Gy per fraction)
	Less than 30 cc	20 Gy (4.0 Gy per fraction)
Prostatic urethra	Maximum point dose within 1mm of target*	42 Gy (8.4 Gy per fraction)
Bladder	Maximum point dose	No more than 105% of prescription dose
	40% of total volume	20 Gy
	5% of total volume	40 Gy
	Highest dose received by 2 cc	<39 Gy
Femoral heads	Less than 10 cc cumulative (both sides)	20 Gy (4.0 Gy per fraction)
Skin	Maximum point dose	15 Gy (3.0 Gy per fraction)
Penile bulb	Maximum dose to 5% of volume	24.8 Gy (4.96 Gy per fraction)
	Mean dose	16 Gy
Sigmoid	Maximum point dose	38 Gy
	Highest dose received by 1 cc	30 Gy

	Highest dose received by 30 cc	25 Gy

*Maximum point dose here refers to the highest dose to 0.035 cc of the target.

5.5. Radiation Therapy Delivery: Patient Set-up and Image Guidance

Patient immobilization will be the same as at simulation (i.e., a custom vacloc bag, alpha-cradle or equivalent immobilization device should be used). The same bladder filling protocol as employed at simulation will be used again, and the patient will be asked to perform Fleet enemas again as well. For patients receiving CT-based SBRT, initial alignment will be based on rigid registration to fiducial markers using planar x-rays. After this initial alignment, a cone beam CT will be obtained to assess OAR geometry. After approval for treatment by an attending radiation oncologist, planar x-rays will be repeated and rigid registration to the fiducial markers will again be performed. Then volumetric modulated arc therapy will be delivered with an estimated total delivery time of <4.5 minutes. No intrafraction motion management will be performed. For patients receiving MRI-based treatment, a 0.35T MRI (1.5 mm isotropic voxel size or better) free-breathing scan will be taken to establish target and OAR geometry at the time of treatment. The pre-treatment MRI will be rigidly registered to the simulation MRI prior to treatment initiation. Alignment will be approved by an attending physician. Once approved, a cine MRI will be obtained at 4 frames per second for the duration of treatment. A 3 mm margin will be placed around the CTV, and if more than 10% of the visualized CTV moves outside this margin, the beam will be held. If the beam is held for >120 second, a manual override will be required to resume treatment. Online adaptive therapy will not be used (NB: initially, it was thought that online adaptive therapy might be used; however, it was deemed nonstandard after the first few patients were treated). Treatments will be delivered every other day, or consecutive days if necessary with all fractions to be delivered within a period not exceeding 14 consecutive chronologic days.

5.6. Specimen Collection for Translational Research

The overall objective of collecting specimens for translational research is to prospectively establish a repository of biospecimens from patients to facilitate current and future hypothesis generated research. Translational research studies integrate the newest research findings into current protocols to investigate important biologic questions. For this protocol, a cheek swab will be collected from each patient, whenever feasible. Cheek swabs will be collected once at any time point, though preferably prior to treatment. All specimens must be labeled with protocol number, patient registration number, and date of specimen collection.

6.0 HORMONAL THERAPY

Hormonal therapy (HT) will be delivered at the discretion of the treating physician. Per the National Comprehensive Cancer Network guidelines,[2] HT is recommended at a duration of 4-6 months for unfavorable intermediate-risk prostate cancer or 12-36 months for high-risk prostate cancer (with 12-36 months in the context of extremely dose-escalated radiotherapy, and 18-36 months for standard dose-escalated radiotherapy). HT generally consists of combined androgen blockade, which is comprised of (a) a luteinizing hormone-releasing hormone agonist (e.g., leuprolide) or a gonadotropin-releasing hormone antagonist (e.g., degarelix) and (b) an oral anti-androgen (e.g., bicalutamide). In combined androgen blockade, the anti-androgen is generally given for one to six months. Given the emerging roles of advanced anti-androgen agents, particularly in high-risk disease,[33] enhanced HT agents may also be given per physician discretion.

7.0 PATIENT ASSESSMENTS AFTER TREATMENT

Patients will be followed clinically after treatment per standard of care. The study calendar (Appendix) includes a summary of the follow-up assessments.

7.1 Toxicity Scoring and Reporting

The study will not be using separate toxicity scales for acute and late radiation adverse events.

7.2.1. Physician-scored toxicity:

All acute and late adverse events from protocol radiation therapy will be reported and scored for severity using the NCI Common Terminology Criteria for Adverse Events (CTCAE) version 4.0.3.

7.2.2. Patient-reported toxicity:

Patient-reported quality of life measures will be collected using several patient-reported outcome instruments.

(a) The Expanded Prostate Cancer Index-26 (EPIC-26) questionnaire will be used to assess changes in the urinary incontinence, urinary obstruction, bowel, sexual function, and hormone/vitality domains.[34] Changes in domain scores at each time point will be classified as minimally important differences or not as previously reported.[1]
(b) The International Prostate Symptom Score (IPSS) questionnaire will be used to assess lower urinary tract symptoms.[35] Scores less than or equal to 7 indicate mild symptoms, scores ranging from 8-19 indicate moderate symptoms, and scores ranging from 20-35 indicate severe symptoms. A single question is included to inquire about quality of life, and the answers range from “delighted” to “terrible” or from 0 to 6. Both changes in total IPSS scores and the total IPSS score at any given time point will be reported.
(c) The International Index of Erectile Function-5 (IIEF-5) or Sexual Health Inventory for Men (SHIM) questionnaire will be used to assess erectile function.[36] The maximum score of 25 indicates maximal erectile function, while scores of 17 or lower indicate erectile dysfunction.[37] Both changes in total SHIM scores and the total SHIM score at any given time point will be reported.

Data will be obtained at baseline (pre-treatment), at 1 month post-SBRT, at 3 months post-SBRT, and then every 3 months for the first year after treatment, and then every 6 months for a minimum of 5 years after treatment (=/- 4 weeks). After 5 years have been elapsed, these will be collected on an annual basis (+/- 4 weeks). Visits after 3 months, or at any timepoint at which the investigator believes it is in the best interest of the subject, these visits may be performed remotely.

7.2. Relevant SBRT-Related Treatment Toxicities

These are the significant and relevant toxicities that are most likely to be related to therapy.

7.3.1. Constitutional symptoms:

Constitutional symptoms that may be attributed to radiation therapy are rare, but include loss of appetite and fatigue. Patients will be seen on a weekly basis while they are receiving radiation therapy per standard of care. Counseling and medications may be prescribed to alleviate these symptoms while the patient is on treatment. It is expected that symptoms will improve and self-resolve 2-4 weeks after completion of therapy.

7.3.2. Radiation proctitis:

Radiation proctitis is due to radiation-induced inflammation of the rectum starting roughly 1 week after treatment. Patients will be seen at least on a weekly basis while they are receiving radiation therapy per standard of care. Counseling, diet and medications may be prescribed to alleviate these symptoms while the patient is on treatment. It is expected that that symptoms will improve and self-resolve within 4-8 weeks after completion of therapy.

7.3.3. Radiation cystitis/urethritis:

Radiation cystitis/urethritis is due to radiation-induced inflammation of the lower urinary tract (bladder/urethra) starting during or within 1-2 weeks after treatment. Patient will be seen on a weekly basis while they are receiving radiation therapy per standard of care. Counseling and medications may be prescribed to alleviate these symptoms while the patient is on treatment. It is expected that that symptoms will improve and self-resolve 4-8 weeks after completion of therapy.

7.3. Measures of Oncologic Efficacy

Follow-up for patients in this study will be consistent with patients managed with definitive radiotherapy for prostate cancer. This follow-up consists of PSA drawn every 3 months for the first year, then every 6 months until 5 years have passed since SBRT, and then once per year subsequently. Data of routine imaging (bone scan, CT or MRI) as clinically indicated will be collected on any patient who presents with any symptoms or PSA progression consistent with cancer recurrence. Recurrences will be managed according to the standard of care after primary radiotherapy for prostate cancer.

Disease Status Definitions:

After study entry, disease evaluations will be made and recorded using the following criteria:

7.1.1. No Evidence of Disease (NED):

No clinical or biochemical (i.e., PSA-based) evidence of disease recurrence

7.1.2. Biochemical Recurrence:
PSA rising above pre-SBRT level and/or continued rise in PSA
7.1.3. Progression of Disease (PD):

Progression of disease will be declared if one or more of the following criteria are met:

Biochemical recurrence

Initiation of salvage therapy

Clinical or radiographic evidence of metastases

7.1.4. Local Progression:

Local progression will be determined by imaging evidence of a recurrence within the prostate fossa, which is proven on biopsy to be recurrent PCa

7.1.5. Distant Failure:
Distant failure will be determined by documented nodal or skeletal metastases as evidenced by imaging
7.1.6. Death from prostate cancer:

Cause of death will be determined by death certificates

Disease-Free Interval Definitions:

The disease-free intervals will be measured from the date of accession to the date of documentation of progression or until the date of death (from other causes)

7.1.7. Time to Biochemical Recurrence:
The date of biochemical recurrence will be defined as the date of a PSA laboratory value that exceeds the pre-SBRT PSA.
7.1.8. Time to Progression:
This will be measured from the date of study entry to the date of whichever occurs first from the following events: biochemical failure, initiation of salvage therapy post-SBRT, clinical or radiographic evidence of tumor recurrence.
7.1.9. Time to Local Progression:
The time to progression will be measured from the date of study entry to the date of documented local progression as determined by imaging evidence of a recurrence followed by histologic confirmation by biopsy.
7.1.10. Time to Distant Failure:
The time to distant failure will be measured from the date of study entry to the date of documented regional nodal recurrence or distant disease relapse. Patients with evidence of biochemical failure, but a negative prostate biopsy, will be considered as distant failure only.
7.1.11. Disease-Specific Survival:
Disease-specific survival will be measured from the date of study entry to the date of death due to prostate cancer. The following will be considered as failure events in assessing disease specific survival:

- Death certified as due to prostate cancer.
- Death from other causes with active malignancy (clinical or biochemical progression).
- Death due to complications of treatment, irrespective of the status of malignancy.

- Death from other causes with previously documented relapse (either clinical or biochemical) but inactive at the time of death will not be considered in disease-specific survival, but will be analyzed.

7.1.12. Overall Survival:

The survival time will be measured from the date of study entry to the date of death. All patients will be followed for survival and effort will be made to document the cause of death.

8.0 STUDY CALENDAR

Procedure	**Pre Study**	**Pre-RT (up to 4 months prior)**	**Baseline Pre-SBRT Day 1**	**On Tx SBRT 5 fx**	**POST TREATMENT VISITS (+/- 4 weeks)***								
					**1 month (+/- 4 wks)**	**3 month (+/- 4 wks)**	**6 month (+/- 4 wks)**	**9 month (+/- 4 wks)**	**12 month (+/- 4 wks)**	**18 month (+/- 4 wks)**	**24 month (+/- 4 wks)**	**Q 6month x 4 yrs 30M 48M 36M 54M 42M 60M (+/- 4 wks)**	**EOS Or Early Term Visit**
Informed Consent	x												
Demographics	x												
Medical History	x												
SOC Physical Exam	x												x
Toxicity assessments / Quality of Life Questionnaires		x**	x**		x	x	x	x	x	x	x	x	x
SOC Non-Contrast Pelvic CT scan***		x											
SOC MRI of the Prostate or Pelvis		x											
SOC PSA Draw****		x				x	x	x	x	x	x	x	x
Translational saliva collection☼			x										
Record Radiation Therapy - 5 fractions				x									

* Follow-up visits can be conducted over the telephone, with remote collection of QOL information
**Toxicity and QOL assessment required either at pre-RT OR pre-SBRT, but not at both time points
*** For patients with PSA <1.0 ng/mL, the treatment planning CT can substitute for a diagnostic CT scan (in this case, the CT simulation should be within 1 month of radiotherapy initiation)
**** These SOC blood samples can be drawn remotely, in the event that the patient is following up outside the UCLA system. In these cases, the lab reports should be provided to the study investigators.
☼ **Whenever feasible, but may be waived**
EOS, end of study; SOC, standard of care

9. DATA REPORTING AND REGULATORY CONSIDERATIONS

9.1. Data Management

The radiation oncology research staff will be responsible for the database records of study patients. The data will be kept on the research coordinator's computer, under password protection, with the patient information de-identified (study patients will be referred by their coded study number). A chart with all the relevant research patient information will be maintained for each patient by the research coordinator, and will be filed in a firewall protected computer. Only the research team (study coordinator, investigators, and project supporting staff) will have the password and key to the data from the study patients.

9.2. Confidentiality

Study data will be maintained in password protected computer files. Only research personnel will have access to this information. All identifiers will be removed. Specimens will be stored under the patient's coded study number. The patient's name or other public identifiers will not be included in any information shared with other investigators. The master key that will identify specific study patients to their coded study number will be kept in a separate password protected file on the research coordinator's computer. Only the study coordinator and the principal investigator will know the password to this file.

10.0 STATISTICAL CONSIDERATIONS

10.1.Study Design and Primary Endpoint

This is a randomized trial that is meant to demonstrate lower rates of acute grade ≥2 GU toxicity following MRI-guided SBRT versus CT-guided SBRT. Specifically, the study is designed to identify a 14% absolute reduction in acute grade ≥2 GU toxicity from 29% to 15%, corresponding to a 52% relative reduction.

10.2. Sample Size and Power Considerations

The best estimates of acute grade ≥2 toxicities following modern SBRT come from the PACE-B trial (acute)[12] and a multi-center SBRT trial run in the United States (late).[21] Overall, one would expect modern SBRT to have acute grade ≥2 GU and GI toxicity rates of 29.1% and 16.0%, and 5-year incidences of late grade ≥2 GU and GI toxicity of 13.3% and 2.0%. Based on a recently reported phase II study, MRI-guided SBRT may lead to acute grade ≥2 GU and GI toxicity rates of 19.8% and 3.0%.[28] Given that these results were based off an early MRI-guided SBRT experience, it is reasonable to anticipate an acute grade ≥2 GU toxicity rate of closer to 15%. However, the doses to be used in the present study are significantly higher than the doses used on the studies providing toxicity estimates for CT-guided and MRI-guided SBRT, and therefore event rates might be higher than anticipated.

The primary endpoint for this study is the incidence of acute grade ≥2 GU toxicity. The current study has been designed to detect a 14% reduction in acute toxicity, from 29% to 15%. An overall sample size of 300 patients (150 per arm) will provide 83.7% power to detect an absolute risk reduction of 14% with a one-sided, two-sample Z-test at a 0.025 significance level. Assuming an accrual of 75-100 patients per year, patients will be accrued over a period of 2.5 years and follow-up will continue for 5 years after the last subject is enrolled. However, the primary endpoint would be available for analysis 90 days after the final patient enrolled completes treatment. Because the effect sizes are hypothetical, we will perform an interim analysis after 100 patients are evaluable for the primary endpoint analysis (i.e., 90 days after SBRT is completed) to allow a revision of the sample size.

Note, the first interim analysis was conducted for 100 patients after they have reached first 90 days after SBRT and data on the incidence of acute grade>=2 GU physician-reported toxicity has been collected, which occurred on August 31, 2021. This analysis yielded a z-test value of -2.59 and one-sided p-value=0.005 when comparing the proportion of acute grade>=2 GU toxicity between the study arms. The proportion of acute grade>=2 GU toxicity in the CT-guided SBRT (reference group) was found to be 24/51=47%. Our study was originally planned to detect a reduction of 14% in acute grade>=2 GU toxicity in the MRI-guided SBRT. Based on the observed data and z-test statistic of the interim analysis and the original design effect size, we thus performed a sample size re-estimation. A total sample size of 154 patients (n=77 per arm) achieves 89% conditional power. Because this is a superiority study in which the intervention arm involves delivery of the same dose of radiation, but without the need for invasive fiducial marker placement or use of additional X-ray imaging for alignment/tracking, and because the interim analysis appeared so favorable, the decision was made on September 9, 2021 to close the study as >154 patients had enrolled.

The study will also evaluate differences in the rate acute grade ≥2 GI toxicity as well as the 5-year cumulative incidences of late grade ≥2 GU and GI toxicity. Other secondary endpoints include 5-year BCRFS (which will be stratified by risk group) and the proportion of fractions delivered that will require online adaptation. Given the assumed toxicity rates, the absolute differences in the secondary toxicity endpoints that could be identified with 80% power, one-sided alpha 2.5% with n=150 patients per arm are provided below

Secondary Endpoint	Assumed Rate in CT-Guided SBRT Arm	Absolute Difference at 80% Power (n=150 per arm)
Acute grade ≥2 GI toxicity	16.0%	8.3%
Late grade ≥2 GU toxicity	13.3%	12.8%
Late grade ≥2 GI toxicity	2.0%	7.5%

10.3. Analysis Populations

Intent-to-Treat (ITT) Population: All patients who are randomized and receive at
least one fraction of SBRT treatment. Analysis of patient-reported outcomes and other secondary efficacy endpoints will be performed on ITT population with a baseline and at least one post-baseline observation.

Safety Population: This population is the subset of the ITT population that actually received the assigned trial intervention

10.4. Statistical Analysis Plan

10.4.1. Analysis of Primary Endpoint:

The primary endpoint is the incidence of acute grade ≥2 GU toxicity on the CTCAE v 4.03 scale. The timeframe for acute toxicity will be restricted to the first 90 days after SBRT, and rates will be reported descriptively. Point estimates as well as the associated 95% C. I.s will be reported.

10.4.2. Analysis of Secondary Endpoints:

Secondary endpoints include the incidence of acute grade ≥2 GI toxicity on the CTCAE v 4.03 scale. The timeframe for acute toxicity will be restricted to the first 90 days after SBRT, and rates will be reported descriptively. Other secondary endpoints include 5-year cumulative incidences of late grade ≥2 GU and GI toxicity based on the CTCAE v 4.03 scale, which will be analyzed using a cumulative incidence framework. The analysis of both acute and late GI toxicity will be stratified for use of hydrogel spacers or not, as these may reduce both acute and late GI toxicity, and by use of nodal radiotherapy or not, if event rate permits this analysis.

Patient-reported outcomes on various quality of life (QOL) instruments will be other secondary endpoints of interest. For the Expanded Prostate Cancer Index-26 (EPIC-26) instrument, these will be represented by changes from baseline in the urinary incontinence, urinary obstruction, bowel, sexual function, and hormone/vitality domains. Changes will be analyzed with respect to whether they represent clinically relevant differences.[1] For the International Prostate Symptom Scores (IPSS) and Sexual Health Inventory for Men (SHIM) QOL indices, the numerical change from baseline, as well as the raw score at any given timepoint, will be extracted and reported descriptively; an absolute change of ≥15 points on IPSS or ≥10 points on the SHIM will be considered a clinically relevant change. Analysis for QOL will be performed using a restricted maximum likelihood (REML)-based mixed models repeated measures (MMRM) approach. The model will include the fixed categorical effects of treatment, time, and treatment-by-time interaction, as well as the other fixed baseline covariates. An unstructured covariance structure will be used to model the within-patient errors. The Kenward-Roger approximation will be used to estimate denominator degrees of freedom. The analysis of both acute and late changes in the bowel domain of the EPIC instrument will be stratified for use of hydrogel spacers or not, as these may reduce both acute and late GI bowel symptoms. The primary endpoint will also be evaluated using a multivariable analysis adjusted for variables including a simultaneous integrated boost, use of nodal radiotherapy, and use of hydrogel spacers.

Five-year BCRFS will be estimated by the Kaplan-Meier method as well as descriptively (mean, standard deviation, median, first and third quartiles, minimum, maximum)., with biochemical recurrence (BCR) defined as serum PSA levels that are 2 ng/mL higher than the nadir PSA achieved after SBRT. Figures showing the Kaplan-Meier estimates will also be presented. Death of any cause will be treated as a competing risk.

10.4.3. Exploratory Analyses:

Several exploratory analyses are planned. The major one will be an analysis of the frequency of beam-holds performed on the MRI-guided arm and associations with the primary outcome. The p-value threshold for significance for these exploratory analyses will be 0.1. Another exploratory comparison will be differences in dosimetric parameters between arms, as well as univariable analyses of predictors of the primary outcome and of grade ≥2 GI toxicity.

<u>10.5. Interim Analysis and Reports:</u>

If the rate of grade 3 or higher adverse gastrointestinal or genitourinary events is higher than 20%, accrual will be halted and the study subjected to careful review. If the rate is higher than 30%, the study will be terminated.

We will follow up all patients closely and do not expect any patients to drop out of the trial except for death from another cause, so censoring due to other reasons would be unlikely. We expect to complete the accrual in 2.5 years. Physician-scored toxicity is the primary endpoint of interest. As described in the sample size consideration section, we will perform an interim analysis for the primary endpoint once 100 patients can be evaluated for this. This will be done to allow a revision of the needed sample size. We will also examine the rate of acute grade 3 or higher adverse gastrointestinal or genitourinary events.

Interim reports will be prepared every six months until the results of the study are published. In general, the interim reports will contain information about patient accrual rate with projected completion dates, status of QA review and compliance rate of treatment per protocol, and the frequencies and severity of toxicity.

11.0 COST CONSIDERATIONS

The proposed treatment approach does not incur additional work-up, procedures, or costs above the standard of care.

12.0 REFERENCES

1. Skolarus TA, Dunn RL, Sanda MG, et al. Minimally important difference for the Expanded Prostate Cancer Index Composite Short Form.
2. NCCN Clinical Practice Guidelines in Oncology: Prostate Cancer. 2015. (Accessed 11/30/2015, at http://www.nccn.org/professionals/physician_gls/pdf/prostate.pdf.)
3. Brenner DJ, Hall EJ. Fractionation and protraction for radiotherapy of prostate carcinoma. International journal of radiation oncology, biology, physics 1999;43:1095-101.
4. Dearnaley D, Syndikus I, Mossop H, et al. Conventional versus hypofractionated high-dose intensity-modulated radiotherapy for prostate cancer: 5-year outcomes of the randomised, non-inferiority, phase 3 CHHiP trial. The Lancet Oncology 2016;17:1047-60.
5. Lee WR, Dignam JJ, Amin MB, et al. Randomized Phase III Noninferiority Study Comparing Two Radiotherapy Fractionation Schedules in Patients With Low-Risk Prostate Cancer. Journal of clinical oncology : official journal of the American Society of Clinical Oncology 2016;34:2325-32.
6. Catton CN, Lukka H, Gu CS, et al. Randomized Trial of a Hypofractionated Radiation Regimen for the Treatment of Localized Prostate Cancer. Journal of clinical oncology : official journal of the American Society of Clinical Oncology 2017;35:1884-90.
7. Hoffman KE, Voong KR, Levy LB, et al. Randomized Trial of Hypofractionated, Dose-Escalated, Intensity-Modulated Radiation Therapy (IMRT) Versus Conventionally Fractionated IMRT for Localized Prostate Cancer.
8. Morgan SC, Hoffman K, Loblaw DA, et al. Hypofractionated Radiation Therapy for Localized Prostate Cancer: An ASTRO, ASCO, and AUA Evidence-Based Guideline.
9. King CR, Freeman D, Kaplan I, et al. Stereotactic body radiotherapy for localized prostate cancer: pooled analysis from a multi-institutional consortium of prospective phase II trials. Radiotherapy and oncology : journal of the European Society for Therapeutic Radiology and Oncology 2013;109:217-21.
10. Widmark A, Gunnlaugsson A, Beckman L, et al. Ultra-hypofractionated versus conventionally fractionated radiotherapy for prostate cancer: 5-year outcomes of the HYPO-RT-PC randomised, non-inferiority, phase 3 trial.
11. Michalski JM, Yan Y Fau - Watkins-Bruner D, Watkins-Bruner D Fau - Bosch WR, et al. Preliminary toxicity analysis of 3-dimensional conformal radiation therapy versus intensity modulated radiation therapy on the high-dose arm of the Radiation Therapy Oncology Group 0126 prostate cancer trial.
12. Brand DH, Tree AC, Ostler P, et al. Intensity-modulated fractionated radiotherapy versus stereotactic body radiotherapy for prostate cancer (PACE-B): acute toxicity findings from an international, randomised, open-label, phase 3, non-inferiority trial.
13. Quon HC, Ong A, Cheung P, et al. Once-weekly versus every-other-day stereotactic body radiotherapy in patients with prostate cancer (PATRIOT): A phase 2 randomized trial.
14. Kishan AU, Dang A, Katz AJ, et al. Long-term Outcomes of Stereotactic Body Radiotherapy for Low-Risk and Intermediate-Risk Prostate Cancer.
15. Kishan AU, Collins SP, Nickols NG. Optimal patient selection for stereotactic body radiotherapy.
16. Siegel R, Naishadham D, Jemal A. Cancer statistics, 2012. CA: a cancer journal for clinicians 2012;62:10-29.
17. Mariotto AB, Yabroff KR, Shao Y, Feuer EJ, Brown ML. Projections of the cost of cancer care in the United States: 2010-2020. Journal of the National Cancer Institute 2011;103:117-28.

18. Laviana AA, Ilg AM, Veruttipong D, et al. Utilizing time-driven activity-based costing to understand the short- and long-term costs of treating localized, low-risk prostate cancer. Cancer 2016;122:447-55.
19. Hodges JC, Lotan Y, Boike TP, Benton R, Barrier A, Timmerman RD. Cost-effectiveness analysis of stereotactic body radiation therapy versus intensity-modulated radiation therapy: an emerging initial radiation treatment option for organ-confined prostate cancer. Journal of oncology practice / American Society of Clinical Oncology 2012;8:e31s-7s.
20. Yu JB, Cramer LD, Herrin J, Soulos PR, Potosky AL, Gross CP. Stereotactic body radiation therapy versus intensity-modulated radiation therapy for prostate cancer: comparison of toxicity. Journal of clinical oncology : official journal of the American Society of Clinical Oncology 2014;32:1195-201.
21. Meier RM, Bloch DA, Cotrutz C, et al. Multicenter Trial of Stereotactic Body Radiation Therapy for Low- and Intermediate-Risk Prostate Cancer: Survival and Toxicity Endpoints.
22. de Crevoisier R, Bayar MA, Pommier P, et al. Daily Versus Weekly Prostate Cancer Image Guided Radiation Therapy: Phase 3 Multicenter Randomized Trial.
23. Dang A, Kupelian PA, Cao M, Agazaryan N, Kishan AU. Image-guided radiotherapy for prostate cancer.
24. Alayed Y, Davidson M, Quon H, et al. Dosimetric predictors of toxicity and quality of life following prostate stereotactic ablative radiotherapy.
25. Pathmanathan AU, Schmidt MA, Brand DH, Kousi E, van As NJ, Tree AC. Improving fiducial and prostate capsule visualization for radiotherapy planning using MRI.
26. Pathmanathan AU, van As NJ, Kerkmeijer LGW, et al. Magnetic Resonance Imaging-Guided Adaptive Radiation Therapy: A "Game Changer" for Prostate Treatment?
27. Lim-Reinders S, Keller BM, Al-Ward S, Sahgal A, Kim A. Online Adaptive Radiation Therapy.
28. Bruynzeel AME, Tetar SU, Oei SS, et al. A Prospective Single-Arm Phase 2 Study of Stereotactic Magnetic Resonance Guided Adaptive Radiation Therapy for Prostate Cancer: Early Toxicity Results.
29. Kishan AU, Lamb JM, Jani SS, Kang JJ, Steinberg ML, King CR. Pelvic nodal dosing with registration to the prostate: implications for high-risk prostate cancer patients receiving stereotactic body radiation therapy. International journal of radiation oncology, biology, physics 2015;91:832-9.
30. O'Neill AG, Jain S, Hounsell AR, O'Sullivan JM. Fiducial marker guided prostate radiotherapy: a review.
31. Hamstra DA, Mariados N, Sylvester J, et al. Continued Benefit to Rectal Separation for Prostate Radiation Therapy: Final Results of a Phase III Trial.
32. Lawton CA, Michalski J, El-Naqa I, et al. RTOG GU Radiation oncology specialists reach consensus on pelvic lymph node volumes for high-risk prostate cancer. International journal of radiation oncology, biology, physics 2009;74:383-7.
33. James ND, de Bono JS, Spears MR, et al. Abiraterone for Prostate Cancer Not Previously Treated with Hormone Therapy.
34. Szymanski KM, Wei JT, Dunn RL, Sanda MG. Development and validation of an abbreviated version of the expanded prostate cancer index composite instrument for measuring health-related quality of life among prostate cancer survivors. Urology 2010;76:1245-50.
35. Barry MJ, Fowler Fj Jr Fau - O'Leary MP, O'Leary Mp Fau - Bruskewitz RC, et al. The American Urological Association symptom index for benign prostatic hyperplasia. The Measurement Committee of the American Urological Association.
36. Rosen RC, Cappelleri JC, Smith MD, Lipsky J, Pena BM. Development and evaluation of an abridged, 5-item version of the International Index of Erectile Function (IIEF-5) as a diagnostic tool for erectile dysfunction. International journal of impotence research 1999;11:319-26.
37. Bianco FJ, Jr., McHone BR, Wagner K, et al. Prevalence of erectile dysfunction in men screened for prostate cancer. Urology 2009;74:89-93.

APPENDICES
