# Supplementary material for: Magnetic Resonance Imaging–Guided vs Computed Tomography–Guided Stereotactic Body Radiotherapy for Prostate Cancer: The MIRAGE Randomized Clinical Trial
Source: JAMA Oncol. 2023 Jan 12;9(3):365–73. doi: 10.1001/jamaoncol.2022.6558 (PMC9857817; doi:10.1001/jamaoncol.2022.6558)
Supplement: Supplement 2. — eTable 1. Details on Treatment Delivery Times eTable 2. Comparison of Dosimetric Parameters Between CT- and MRI-Guided Arms eTable 3. Sensitivity Analysis for Differences in Acute Genitourinary and Gastrointestinal Toxicity Including Patients Not Who Were Analyzed But Not Evaluable eTable 4. Multivariate Analysis for Acute Grade ≥2 Genitourinary Toxicities eTable 5. Differences in Grade ≥2 Genitourinary and Gastrointestinal Toxicities Stratified by Baseline International Prostate Symptom Score (IPSS) eTable 6. Differences in Grade ≥2 Genitourinary and Gastrointestinal Toxicities Stratified by Baseline Prostate Volume eTable 7. Acute Grade ≥2 Genitourinary and Gastrointestinal Toxicity Rates in Selected Other Stereotactic Body Radiotherapy Studies eFigure 1. Longitudinal Changes in Urinary Irritative/Obstructive and Total Urinary Expanded Prostate Cancer Index Composite-26 (EPIC-26) Scores eFigure 2. Proportions of Patients With Clinically Relevant Declines in Expanded Prostate Cancer Index Composite-26 (EPIC-26) Scores Urinary Subdomains eFigure 3. Longitudinal Changes in Total International Prostate Symptom Score (IPSS) and IPSS Quality-of-Life Scores eFigure 4. Longitudinal Changes in Sexual and Hormonal Expanded Prostate Cancer Index Composite (EPIC-26) Scores eFigure 5. Proportions of Patients With Clinically Relevant Declines in Sexual and Hormonal Expanded Prostate Cancer Index Composite (EPIC-26) Scores eReferences [file jamaoncol-e226558-s002.pdf]

Supplemental Online Content

Kishan AU, Ma TM, Lamb JM, et al. Magnetic resonance imaging-guided vs computed tomography-guided stereotactic body radiotherapy for prostate cancer: the MIRAGE randomized clinical trial. *JAMA Oncol*. Published online January 12, 2023.
doi:10.1001/jamaoncol.2022.6558

eTable 1. Details on Treatment Delivery Times
eTable 2. Comparison of Dosimetric Parameters Between CT- and MRI-Guided Arms
eTable 3. Sensitivity Analysis for Differences in Acute Genitourinary and Gastrointestinal Toxicity Including Patients Not Who Were Analyzed But Not Evaluable
eTable 4. Multivariate Analysis for Acute Grade ≥2 Genitourinary Toxicities
eTable 5. Differences in Grade ≥2 Genitourinary and Gastrointestinal Toxicities Stratified by Baseline International Prostate Symptom Score (IPSS)
eTable 6. Differences in Grade ≥2 Genitourinary and Gastrointestinal Toxicities Stratified by Baseline Prostate Volume
eTable 7. Acute Grade ≥2 Genitourinary and Gastrointestinal Toxicity Rates in Selected Other Stereotactic Body Radiotherapy Studies
eFigure 1. Longitudinal Changes in Urinary Irritative/Obstructive and Total Urinary Expanded Prostate Cancer Index Composite-26 (EPIC-26) Scores
eFigure 2. Proportions of Patients With Clinically Relevant Declines in Expanded Prostate Cancer Index Composite-26 (EPIC-26) Scores Urinary Subdomains
eFigure 3. Longitudinal Changes in Total International Prostate Symptom Score (IPSS) and IPSS Quality-of-Life Scores
eFigure 4. Longitudinal Changes in Sexual and Hormonal Expanded Prostate Cancer Index Composite (EPIC-26) Scores
eFigure 5. Proportions of Patients With Clinically Relevant Declines in Sexual and Hormonal Expanded Prostate Cancer Index Composite (EPIC-26) Scores
eReferences

This supplemental material has been provided by the authors to give readers additional information about their work.

© 2023 Kishan AU et al. *JAMA Oncology.*

eTable 1. Details on Treatment Delivery Times

	CT-guidance (n=77)	MRI-guidance (n=79)
Number of Arcs		
2	49 (64%)	
3	1 (1%)	
4	27 (35%)	
Number of Beams (median, IQR)		15 (15-17)
Post-Imaging Delivery Time (per fraction, second)		
Median (IQR)	232 (198-272)	1133.4 (1009.0-1289)

IQR, interquartile range

© 2023 Kishan AU et al. *JAMA Oncology.*

eTable 2. Comparison of Dosimetric Parameters Between CT- and MRI-Guided Arms

Parameter	CT-Guidance	MRI-Guidance	*P* value
CTV			
Volume	55.9 (44.2-66.9)	50.6 (37.7-60.6)	0.5
PTV			
**Volume**	**102.1 (82.5-119.6)**	**70.5 (52.6-87.3)**	**<0.001**
V_{40Gy}	95% (95%-95%)	95% (94.1-95.0%)	0.37
**V_{42Gy}**	**5.4% (0.4-14.9%)**	**18.6% (12.2-25.5%)**	**<0.001**
Bladder			
$D_{0.035cc}$	41.2 (40.8-41.8)	41.1 (40.6-41.5)	0.08
**V_{40Gy}**	**0.7 (0.2-1.6)**	**0.3 (0.1-0.44)**	**0.001**
**V_{39Gy}**	**3.7 (2.6-6.1)**	**1.9 (0.9-3.6)**	**<0.001**
V_{20Gy}	10.4 (7.7-17.5)	12.3 (5.9-18.0)	0.51
Urethra DMax	41.6 (41.0-41.9)	41.7 (41.5-42.1)	0.06
Rectum			
$D_{0.035cc}$	40.0 (37.3-41.1)	40.4 (34.4-41.1)	1.0
V_{40Gy}	0.05 (0.0-0.8)	0.2 (0-0.9)	0.13
V_{38Gy}	0.5 (0.03-1.4)	0.8 (0.-1.6)	0.64
V_{36Gy}	1.5 (0.3-3.6)	2.5 (0.03-4.4)	0.31
V_{32Gy}	3.4 (1.3-6.3)	5.0 (0.3-7.2)	0.40
V_{20Gy}	14.1 (9.0-20.0)	18.2 (9.0-22.3)	0.33
**Anal Canal**			
**$D_{0.035cc}$**	**12.3 (10.2-16.4)**	**24.0 (18.9-28.0)**	**<0.001**
**V_{20Gy}**	**0.0 (0.0-0.0)**	**0.1 (0.02-0.3)**	**<0.001**
Small Bowel			
$D_{0.035cc}$	1.7 (0.9-6.5)	1.4 (1.0-10.3)	0.91
V_{20Gy}	0.0 (0.0-0.0)	0.0 (0.0-0.0)	0.84
Penile Bulb			
$V_{24.8Gy}$	0 (0-0)	0 (0-0)	0.27
Mean Dose	4.0 (2.6-4.9)	2.7 (2.3-4.5)	0.16

P values obtained using Wilcoxon rank-sum test
All values are reported as median Gy or cc (interquartile range).
$D_{x\ cc}$ refers to maximum dose received by x cc of structure in question; $V_{x\ Gy}$ refers to the volume of structure in question receiving x Gy

© 2023 Kishan AU et al. *JAMA Oncology.*

eTable 3. Sensitivity Analysis for Differences in Acute Genitourinary and Gastrointestinal Toxicity Including Patients Not Who Were Analyzed But Not Evaluable

	CT-guidance	MRI-guidance	*P* value
Grade ≥2 Genitourinary	42.7% (31-.6-54.7)	25.3 % (16.2-36.4)	0.02
Grade ≥2 Gastrointestinal	10.4% (4.6-19.5)	1.3% (0.03-6.7)	0.02

This "extreme" scenario assigns the patient on the MRI-guidance arm as having experienced grade 2 genitourinary and grade 2 gastrointestinal toxicity and assigns the patient on the CT-guidance arm as having experienced no toxicity. Numbers tabulated are the estimate and the 95% confidence interval.

© 2023 Kishan AU et al. *JAMA Oncology.*

eTable 4. Multivariate Analysis for Acute Grade ≥2 Genitourinary Toxicities

**Parameter**	**OR (95% CI)**	***P* value**
**Trial Arm (MRI-guidance vs CT-guidance)**	**0.4 (0.2, 0.9)**	**0.02**
**Age (1-unit increase)**	**1.1 (1.0, 1.2)**	**0.007**
Baseline IPSS (1-unit increase)	1.1 (1.0, 1.1)	0.1
Rectal Spacer (Yes vs No)	1.0 (0.5, 2.2)	*1.0*
Pelvic LN Radiation (Yes vs No)	0.5 (0.2, 1.2)	0.1
GTV Boost (Yes vs No)	1.7 (0.7, 4.4)	0.3
Prostate Gland Volume (1-unit increase)	1.0 (1.0, 1.0)	0.9
Area under ROC curve (95% CI)	0.7 (0.6, 0.8)	n/a

MRI, Magnetic Resonance Imaging; CT, Computed Tomography; IPSS, International Prostate Symptom Score; LN, lymph node; GTV, gross tumor volume; OR, odds ratio; ROC, receiver operating characteristic; CI, confidence interval

© 2023 Kishan AU et al. *JAMA Oncology.*

eTable 5. Differences in Grade ≥2 Genitourinary and Gastrointestinal Toxicities Stratified by Baseline International Prostate Symptom Score (IPSS)

Endpoint	Statistic	CT	MRI)	*P* value
**IPSS ≤15**		**(n=63)**	**(n=68)**	
GU Toxicity ≥2	n (%)	26 (41.3%)	17 (25.0%)	**0.05**
	95% CI	29.0 , 54.4	15.3 , 37.0	
GI Toxicity ≥2	n (%)	5 (7.9%)	0 (0.0%)	**0.02**
	95% CI	2.6 , 17.6	0 , 5.3	
**IPSS >15**		**(n=13)**	**(n=10)**	
GU Toxicity ≥2	n (%)	7 (53.9%)	2 (20.0%)	0.2
	95% CI	25.1 , 80.8	2.5 , 55.6	
GI Toxicity ≥2	n (%)	3 (23.1%)	0 (0%)	0.2
	95% CI	5.04 , 53.8	0 , 30.9	

P values obtained using Chi-square test and Fisher's exact test as appropriate. p-interaction >0.1

© 2023 Kishan AU et al. *JAMA Oncology.*

eTable 6. Differences in Grade ≥2 Genitourinary and Gastrointestinal Toxicities Stratified by Baseline Prostate Volume

Endpoint	Statistic	CT	MRI)	*P* value
**Prostate Volume ≤50 cc**		**(n=49)**	**(n=55)**	
GU Toxicity ≥2	n (%)	24 (48.98%)	12 (21.82%)	**0.004**
	95% CI	34.42 , 63.66	11.81 , 35.01	
GI Toxicity ≥2	n (%)	6 (12.24%)	0 (0%)	**0.009**
	95% CI	4.63 , 24.77	0 , 6.49	
**Prostate Volume >50 cc**		**(n=27)**	**(n=23)**	
GU Toxicity ≥2	n (%)	9 (33.33%)	7 (30.43%)	1.0
	95% CI	16.52 , 53.96	13.21 , 52.92	
GI Toxicity ≥2	n (%)	2 (7.41%)	0 (0%)	0.5
	95% CI	0.91 , 24.29	0 , 14.82	

P value obtained using Chi-square test and Fisher's exact test as appropriate. p-interaction >0.1

© 2023 Kishan AU et al. *JAMA Oncology.*

eTable 7. Acute Grade ≥2 Genitourinary and Gastrointestinal Toxicity Rates in Selected Other Stereotactic Body Radiotherapy Studies

Study	PTV Dose	EQD_2 $\alpha/\beta=2$	EQD_2 $\alpha/\beta=10$	Margins	Acute Grade≥2 GU Toxicity	Acute Grade ≥2 GI Toxicity
Jackson *et al.*[1]	7.4 Gy x 5	77 Gy	53.7 Gy	3 mm	23%	4%
Fuller *et al.*[2]	9.5 Gy x 4	95 Gy	61.8 Gy	2mm/0mm posteriorly	35.1%	6.9%
Meier *et al.*[3]	7.25 Gy x 5	74 Gy	52.1 Gy	5 mm/3 mm	26%	8.1%
Brand *et al.*[4]	7.25 Gy x 5	74 Gy	52.1 Gy	4-5 mm/3-5mm	29.2%	14.9%
Bruynzeel *et al.*[5]	7.25 Gy x 5	74 Gy	52.1 Gy	3 mm	23.8%	5.0%
MIRAGE, CT-Guidance	8 Gy x 5	88 Gy	60 Gy	4 mm	43.4%	10.5%
MIRAGE, MRI-Guidance	8 Gy x 5	88 Gy	60 Gy	2 mm	24.4%	0%

EQD_2, equivalent dose in 2 Gy fractions; GI, gastrointestinal; GU, genitourinary; PTV, planning target volume

© 2023 Kishan AU et al. *JAMA Oncology.*

eFigure 1. Longitudinal Changes in Urinary Irritative/Obstructive and Total Urinary Expanded Prostate Cancer Index Composite-26 (EPIC-26) Scores

P values determined by the Mann-Whitney test.

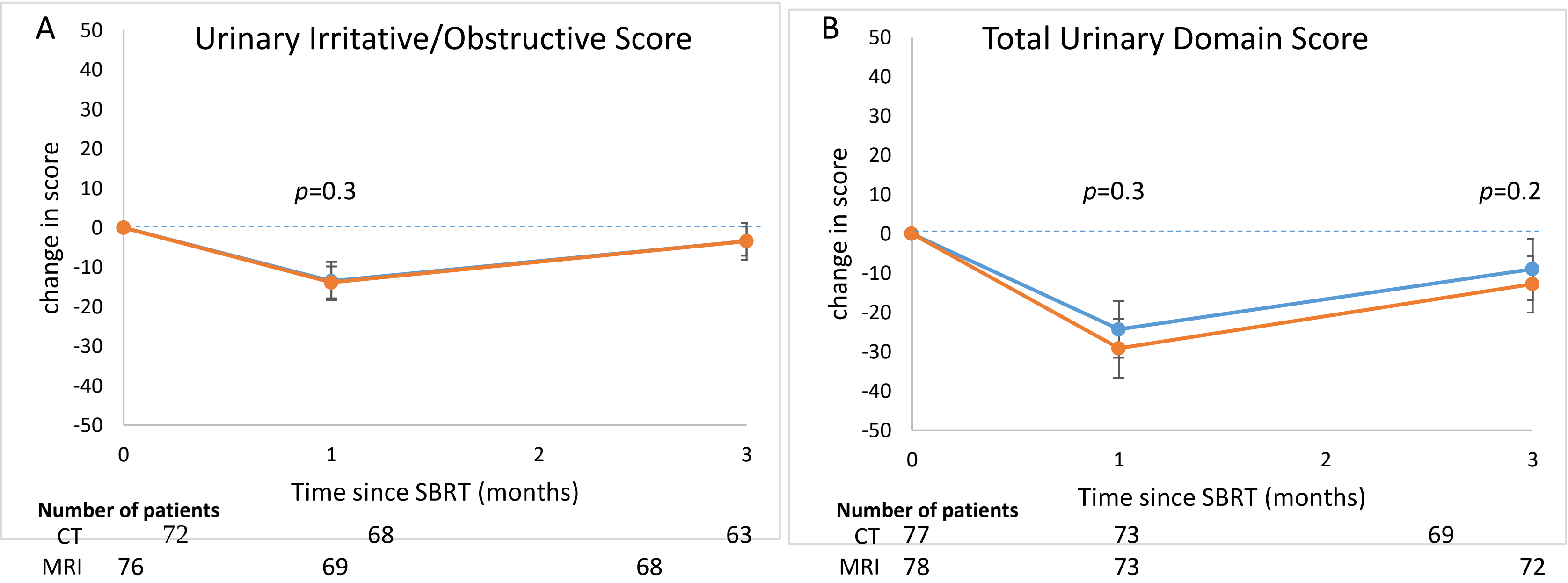

© 2023 Kishan AU et al. *JAMA Oncology.*

eFigure 2. Proportions of Patients With Clinically Relevant Declines in Expanded Prostate Cancer Index Composite-26 (EPIC-26) Scores Urinary Subdomains

Thresholds for clinically relevant declines were ≥18 points for urinary incontinence and ≥14 points for urinary irritative/obstructive subdomains. *P* values determined by chi-square test.

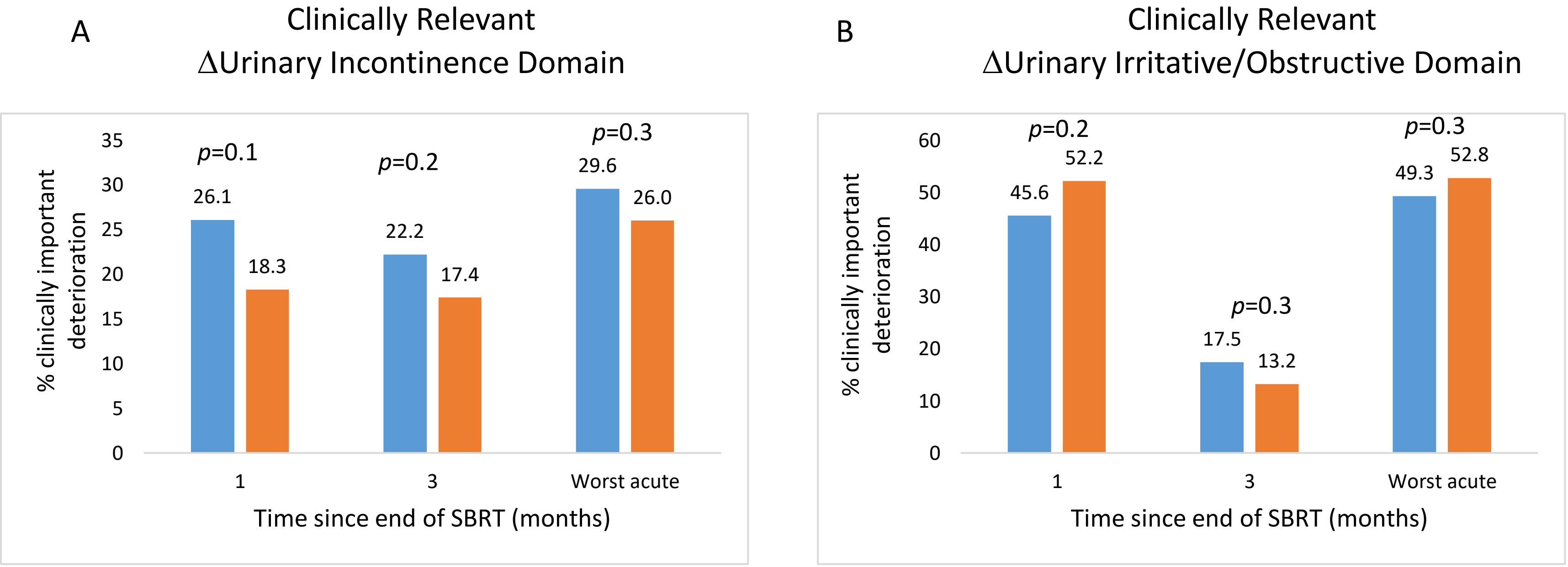

© 2023 Kishan AU et al. *JAMA Oncology.*

eFigure 3. Longitudinal Changes in Total International Prostate Symptom Score (IPSS) and IPSS Quality-of-Life Scores

P values determined by the Mann-Whitney test.

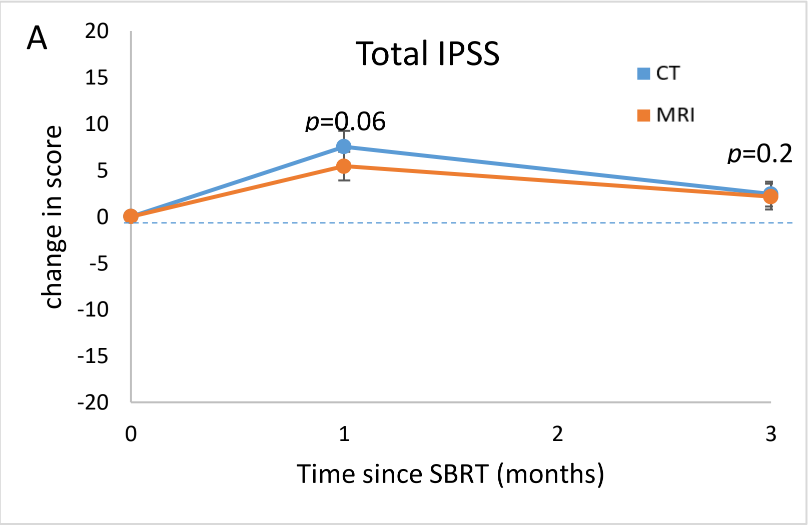

Number of patients

CT	77	72	70
MRI	79	74	73

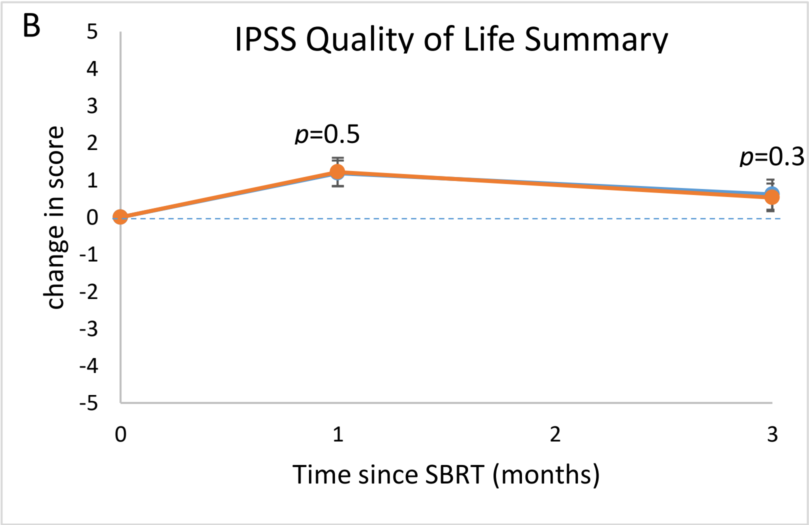

Number of patients

CT	77	70	69
MRI	79	74	71

© 2023 Kishan AU et al. *JAMA Oncology.*

eFigure 4. Longitudinal Changes in Sexual and Hormonal Expanded Prostate Cancer Index Composite (EPIC-26) Scores

P values determined by the Mann-Whitney test. Analysis restricted to men not receiving androgen deprivation therapy.

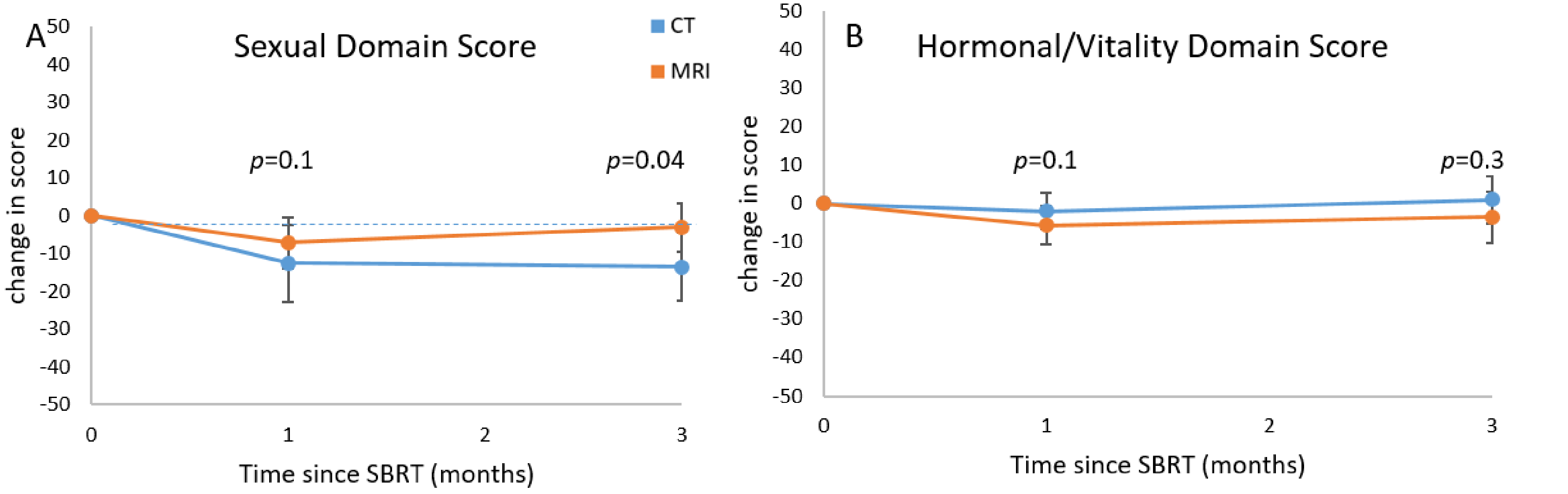

Number of patients			
CT	20	20	16
MRI	30	27	25

Number of patients			
CT	20	20	16
MRI	30	28	24

© 2023 Kishan AU et al. *JAMA Oncology.*

eFigure 5. Proportions of Patients With Clinically Relevant Declines in Sexual and Hormonal Expanded Prostate Cancer Index Composite (EPIC-26) Scores

Thresholds for clinically relevant declines were ≥24 points the sexual domain and ≥12 points for the hormonal/vitality domain score. *P* values determined by chi-square test. Analysis restricted to men not receiving androgen deprivation therapy.

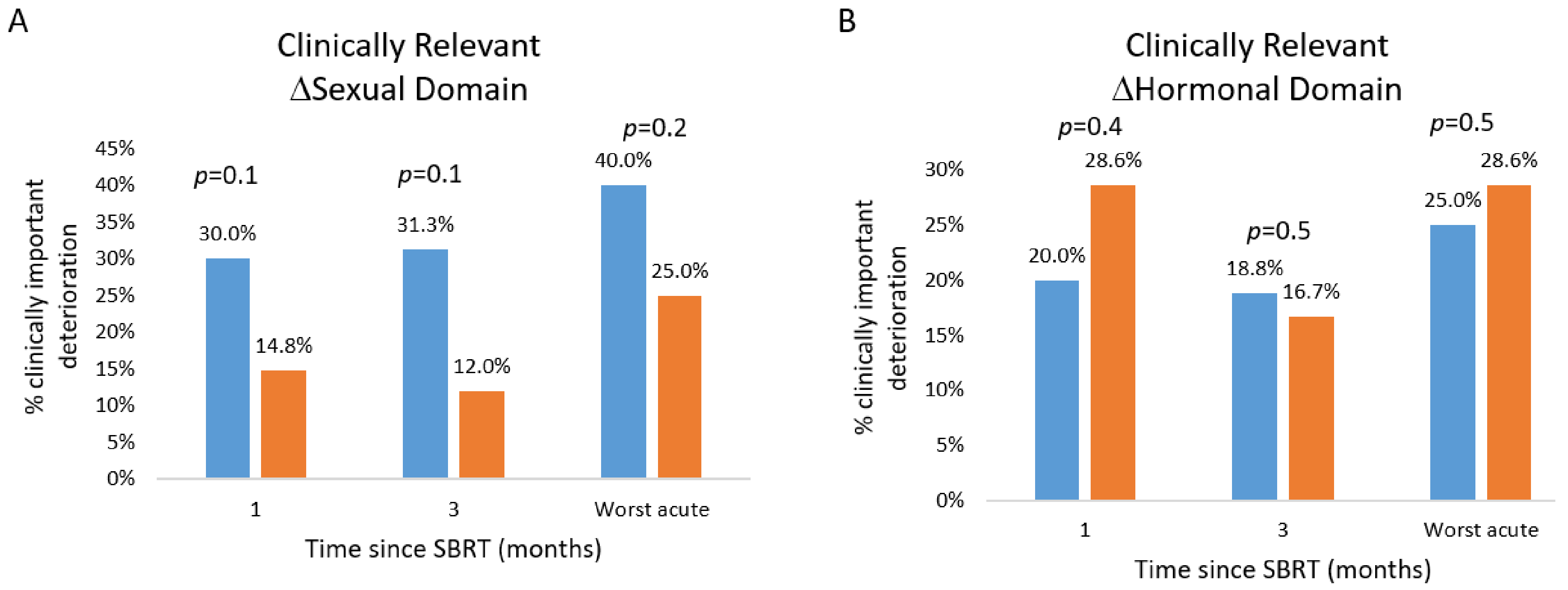

© 2023 Kishan AU et al. *JAMA Oncology.*

eReferences

1. Jackson WC, Dess RT, Litzenberg DW, et al. A multi-institutional phase 2 trial of prostate stereotactic body radiation therapy (SBRT) using continuous real-time evaluation of prostate motion with patient-reported quality of life. *Pract Radiat Oncol* 2018; **8**(1): 40-7.
2. Fuller DB, Falchook AD, Crabtree T, et al. Phase 2 Multicenter Trial of Heterogeneous-dosing Stereotactic Body Radiotherapy for Low- and Intermediate-risk Prostate Cancer: 5-year Outcomes. *Eur Urol Oncol* 2018; **1**(6): 540-7.
3. Meier RM, Bloch DA, Cotrutz C, et al. Multicenter Trial of Stereotactic Body Radiation Therapy for Low- and Intermediate-Risk Prostate Cancer: Survival and Toxicity Endpoints. *Int J Radiat Oncol Biol Phys* 2018; **102**(2): 296-303.
4. Brand DH, Tree AC, Ostler P, et al. Intensity-modulated fractionated radiotherapy versus stereotactic body radiotherapy for prostate cancer (PACE-B): acute toxicity findings from an international, randomised, open-label, phase 3, non-inferiority trial. *Lancet Oncol* 2019; **20**(11): 1531-43.
5. Bruynzeel AME, Tetar SU, Oei SS, et al. A Prospective Single-Arm Phase 2 Study of Stereotactic Magnetic Resonance Guided Adaptive Radiation Therapy for Prostate Cancer: Early Toxicity Results. *Int J Radiat Oncol Biol Phys* 2019; **105**(5): 1086-94.

© 2023 Kishan AU et al. *JAMA Oncology.*
