# Supplementary material for: Magnetic Resonance Imaging–Guided vs Computed Tomography–Guided Stereotactic Body Radiotherapy for Prostate Cancer: The MIRAGE Randomized Clinical Trial
Source: JAMA Oncol. 2023 Jan 12;9(3):365–73. doi: 10.1001/jamaoncol.2022.6558 (PMC9857817; doi:10.1001/jamaoncol.2022.6558)
Supplement: Supplement 3. — Data Sharing Statement [file jamaoncol-e226558-s003.pdf]

Data Sharing Statement

Kishan. Magnetic Resonance Imaging-Guided vs Computed Tomography-Guided Stereotactic Body Radiotherapy for Prostate Cancer. *JAMA Oncol*. Published January 12, 2023. doi:10.1001/jamaoncol.2022.6558

Data
Data available: No

Additional Information
Explanation for why data not available: Data from MIRAGE will not be available for data sharing until all endpoints (including the secondary endpoints related to long-term outcomes) have been analyzed.
